# Preclinical Trials for Prevention of Tumor Progression of Hepatocellular Carcinoma by LZ-8 Targeting c-Met Dependent and Independent Pathways

**DOI:** 10.1371/journal.pone.0114495

**Published:** 2015-01-21

**Authors:** Jia-Ru Wu, Chi-Tan Hu, Ren-In You, Pei-Ling Ma, Siou-Mei Pan, Ming-Che Lee, Wen-Sheng Wu

**Affiliations:** 1 Institute of Medical Sciences, Tzu Chi University, Hualein, Taiwan; 2 Department of Laboratory Medicine and Biotechnology, College of Medicine, Tzu Chi University, Hualein, Taiwan; 3 Research Centre for Hepatology, Buddhist Tzu Chi General Hospital and Department of Internal Medicine Tzu Chi University, Hualien, Taiwan; 4 Department of Surgery, Buddhist Tzu Chi General Hospital, School of Medicine, Tzu Chi University, Hualien, Taiwan; University of Medicine, Greifswald, Germany

## Abstract

Hepatocellular carcinoma (HCC) is among the most lethal cancers. Mounting studies highlighted the essential role of the HGF/c-MET axis in driving HCC tumor progression. Therefore, c-Met is a potential therapeutic target for HCC. However, several concerns remain unresolved in c-Met targeting. First, the status of active c-Met in HCC must be screened to determine patients suitable for therapy. Second, resistance and side effects have been observed frequently when using conventional c-Met inhibitors. Thus, a preclinical system for screening the status of c-Met signaling and identifying efficient and safe anti-HCC agents is urgently required. In this study, immunohistochemical staining of phosphorylated c-Met (Tyr1234) on tissue sections indicated that HCCs with positive c-Met signaling accounted for approximately 46% in 26 cases. Second, many patient-derived HCC cell lines were established and characterized according to motility and c-Met signaling status. Moreover, LZ8, a medicinal peptide purified from the herb Lingzhi, featuring immunomodulatory and anticancer properties, was capable of suppressing cell migration and slightly reducing the survival rate of both c-Met positive and negative HCCs, HCC372, and HCC329, respectively. LZ8 also suppressed the intrahepatic metastasis of HCC329 in SCID mice. On the molecular level, LZ8 suppressed the expression of c-Met and phosphorylation of c-Met, ERK and AKT in HCC372, and suppressed the phosphorylation of JNK, ERK, and AKT in HCC329. According to receptor array screening, the major receptor tyrosine kinase activated in HCC329 was found to be the epidermal growth factor receptor (EGFR). Moreover, tyrosine-phosphorylated EGFR (the active EGFR) was greatly suppressed in HCC329 by LZ8 treatment. In addition, LZ8 blocked HGF-induced cell migration and c-Met-dependent signaling in HepG2. In summary, we designed a preclinical trial using LZ8 to prevent the tumor progression of patient-derived HCCs with c-Met-positive or -negative signaling.

## Introduction

Liver cancer is the sixth most common and third most deadly cancer worldwide [1]. Hepatocellular carcinoma (HCC) is the most common type of liver cancer, accounting for 83% of all cases [2]. Diverse pathological mechanisms, such as hepatitis B and hepatitis C viral infection and alcohol or aflatoxin B1 exposure, trigger the development and progression of HCC [3]. Generally, patients with early-stage HCC can receive resection or locoablative therapy, whereas those with multifocal intrahepatic tumors may benefit from transarterial chemoembolization [4,5]. Chemotherapies targeting aberrant molecular pathways involved in HCC have been developed for advanced HCC, which is not feasible for locoregional therapy. Over the past decade, sorafenib, a multikinase inhibitor featuring antiproliferative and proapoptotic properties, has been determined to be the most promising agent for HCC target therapy [6–8]. However, the overall outcomes are far from satisfactory, and the improved overall survival is less than 1 year [9]. Moreover, the acquired resistance to and side effects from sorafenib have drawn attention [10]. An explanation for these drawbacks is the genetic heterogeneity of HCC that leads to the primary resistance to sorafenib. Moreover, because metastatic spreads are responsible for the poor prognosis of most patients with HCC [11,12], the limited response of HCC to antiproliferative drugs such as sorafenib is expected. However, an effective therapy targeting the molecular pathway leading to the tumor metastasis of HCC has not been firmly established.

Tumor metastasis, one of the most complicated pathological processes is initiated by epithelial mesenchymal transition (EMT), migration and invasion of the primary tumor, followed by intravasation, extravasation, and colonization at the metastatic loci [13]. Within the tumor microenvironment, the primary tumor may interact with stromal and inflammatory cells, leading to the secretion of numerous growth factors and cytokines, including hepatocyte growth factor (HGF) [13–16], epidermal growth factor (EGF), and transforming growth factor-β [17]. These soluble factors can induce metastatic changes of primary tumors [14], and therefore may be collectively called metastatic factors. Blocking the molecular pathway mediating the actions of these factors is a promising strategy for inhibiting HCC progression.

Among the metastatic factors, the scatter factor HGF was highlighted to be involved in the progression of cancer [18], including HCC. The receptor tyrosine kinase (RTK) of HGF, c-Met, which is a prototypic member of the RTK family, is involved in diverse cellular responses such as motogenesis and morphogenesis. In HCC, c-Met may be activated in an autocrine fashion as evidenced by high levels of intracytoplasmic HGF [19]. Moreover, high HGF level in serum and deregulated expression of c-Met in HCCs are closely associated with early recurrence [20] and patients with high c-Met expressing HCCs usually have shorter 5-year survival rate after curative surgical resection [19–22]. In addition, a group of HCCs (27%) with a c-Met-induced transcriptional signature was characterized by a higher rate of vascular invasion [23]. In vitro studies have also revealed the effects of HGF on metastatic changes of HCC, including EMT, migration, and invasion [24–26]. Therefore, HGF-c-Met signaling is currently the most promising therapeutic target for preventing HCC progression [2, 27–29].

The binding of HGF to c-Met induces the autophosphorylation of its cytoplasmic domain, followed by the recruitment of upstream regulators, such as Gab, Grb2, and PI3K, that activate downstream mitogen activated protein kinase (MAPK) and AKT signaling [30–32]. In addition, c-Met can be activated independently of HGF by interacting with the EGF receptor, cell attachment or an alternate ligand des-γ-carboxy prothrombin [2].

During the past decades, numerous small molecular c-Met inhibitors have been designed for c-Met-based target therapy. Currently, at least 17 c-Met inhibitors, including JNJ-38877605, GEN-203, and ARQ197, are under clinical evaluation [33]. However, several concerns in targeting the HGF-c-Met axis must be addressed. First, c-Met overexpression has been observed in only 20% to 48% of human HCC cases [21]. For HCC with negative c-Met signaling, c-Met-based target therapy may be ineffective. Secondly, early clinical trials have revealed unexpectedly limited benefits of c-Met inhibition [27], and the side effects caused by c-Met inhibitors, including anemia, neutropenia, and liver and bone marrow toxicity [34,35] have been observed frequently. Thus, searching efficient and safe antagonists that can block critical molecular pathways, either c-Met-dependent or -independent, is critical for preventing HCC progression.

One promising anti-HCC agent is the medicinal peptide LZ8 (also known as Lingzhi or Reishi), which is purified from the Chinese herbal drug Ganoderma lucidium [36]. In previous studies, LZ8 was found to be an immunomodulatory adjuvant that enhanced the efficacy of cancer DNA vaccines by activating dendritic cells [37, 38]. More recently, the prominent anticancer capability of LZ8 was highlighted. LZ8 may suppress proliferation of breast cancer [39] and lung cancer [40, 41] and retard cell migration of cervical cancer [42]. The molecular mechanisms for the antitumor activity of LZ8 have been intensively studied. For example, LZ8 may stabilize p53 and increase the CDK inhibitor p21 [41], leading to cell cycle arrest of lung cancer cell. In addition, LZ8 can repress telomerase activity [43] in lung adenocarcinoma cells. Regarding intracellular signaling, LZ8 may suppress the protein kinase C-dependent pathway [44] involved in cancer progression triggered by HGF-c-Met [45]. Currently, whether LZ8 can suppress HCC progression or influences c-Met-dependent signaling remains un-investigated in preclinical trials.

In this study, we improved the preclinical trials for preventing HCC progression in several aspects. First, to validate the feasibility of c-Met targeting, we screened active c-Met (phosphorylated c-Met; p-c-Met) in HCC tissues. Moreover, using patient-derived HCCs as target cells, we noted that LZ8 can suppress HCC tumor progression, associated with inhibiting the activity of critical signal molecules involved in c-Met- or non-c-Met-dependent pathways.

## Materials and Methods

### Cell lines, hepatocellular carcinoma tissue collection, and chemicals

Human hepatoma cell HepG2 was purchased from the Bioresource Collection and Research Center (Hsinchu, Taiwan). HCC tissues were collected during surgery performed at Tzu Chi Hospital with patient’s consents, which have been approved by the Research Ethics Committee in Buddhist Tzu Chi General Hospital (IRB 101–62). The tissues were snap frozen at −80°C before being harvested for Western blotting or sectioning for immunohistochemical analysis. HGF and JNJ-38877605 were obtained from Peprotech (Rocky Hill, NJ, USA) and MedKoo (Chapel Hilll, NC, USA), respectively. SP600125, PD98059, AG1748, and SU5416 were acquired from Calbiochem (Darmstadt, Germany). Antibodies for c-Met, p-c-Met, phosphorylated JNK (p-JNK), phosphorylated ERK (p-ERK), phosphorylated AKT (p-AKT), phosphorylated paxillin (p-paxillin S178), GAPDH, and ERK were obtained from Santa Cruz Biotechnology, Inc. (California, CA, USA). LZ8 was purchased from the Yeastern Company (Taipei, Taiwan) and prepared using recombinant protein technology in a yeast system. Briefly, DNA sequence encoding LZ8 was cloned and expressed in *Saccharomyces cerevisiae*. Cells expressing LZ8 were disrupted and centrifuged, and the supernatant was passed through molecular sieves to obtain proteins between 10 kDa and 100 kDa large. The filtrate was further purified using Superdex 75 columns (GE Healthcare) and the purity was determined by HPLC.

### Establishing patient-derived hepatocellular carcinoma cell lines

Clinically derived HCC cell lines were established from parts of HCC tissues obtained from surgery with patient’s consents, which have been approved by Buddhist Tzu Chi general hospital Research Ethic Committee (IRB 101–62). All patients signed informed consent forms (with partially hidden names and personal information), a few of which are presented in S1 Materials. The patient-derived cell lines were established according to standard procedures. Briefly, HCC tissues were pretreated with collagenase and cultivated on the mitomycin C-treated NIH3T3 feeder layer for 4 to 6 passages to select the HCC cell lines. Homogenous HCC cell populations were obtained and the sustained proliferation ability (over 20 passages) and metastatic potentials were tested in vitro and in vivo. The characteristics of the HCC tumor cell lines were validated by detecting HCC tumor makers such as Glypican 3 (GCP3) [46], after more than 40 passages.

### Immunohistochemistry

Immunohistochemistry (IHC) for HGF and p-c-Met was performed according to the standard protocols established by the Research Centre for Hepatology at Tzu Chi Hospital.

### Cell proliferation assay

The cells were cultivated in 24-well plates. After appropriate treatments, the cells were counted with hemocytometer every 24 h for 72 h. The doubling time of cells was calculated using the following equation: Time (h)/Generation, where Time is the time from cell seeding to cell counting and Generation is the generation of cell replication and was equal to log N1-log N0/log2 (N0 = number of cells seeded initially; N1 = number of cells counted at a specific time point after seeding).

### Cell cycle analysis

HCC cells were harvested by trypsin detachment and washed in ice-cold, phosphate-buffered saline (PBS). After centrifugation and supernatant removal, the cells were fixed using ice-cold 70% ethanol for 24 h. The cells were then washed with PBS and incubated with RNase A (Sigma-Aldrich; 0.5 mg/mL) and propidium iodide (Sigma-Aldrich; 1 mg/mL) at room temperature for 15 min. The red (FL3) fluorescence of the stained cells was analyzed using a Beckman Coulter Gallios flow cytometer. The percentage of cells in the G1, S, and G2/M phases of the cell cycle was analyzed using Kaluza 1.1 software (Beckman Coulter).

### Wound healing migration assay

The cells were cultivated in 24-well plates with a wound healing culture insert until confluence, and then serum starved for 24 h, after which the culture insert was removed. After appropriate treatments of the cells, images were obtained using phase contrast microscopy. Quantitation of cell motility was performed by counting the cells migrating into the blanking area using Image J software.

### Transwell migration assay

Cells were seeded on a 24-well transwell migration insert (Nalge Nunc International, Rochester, NY, USA) in a complete medium for 24 h. After appropriate treatments, cells that had migrated to the underside of the insert membrane were stained with 0.3% crystal violet. The cells on the topside of the insert membrane were rubbed with a cotton swab. The migrated cells on the underside were imaged using phase contrast microscopy with 200× magnification.

### Western blot

Western blots were performed according to our previous study [45]. The band intensities on the blots were quantified using ImageJ software.

### Establishing hepatocellular carcinoma metastasis in SCID mice

Orthotropic transplantation was performed for tumor inoculation. Initially, approximately 1 × 10^7^ cells were injected subcutaneously into the right flank of the mice, and the signs of tumor development were then monitored daily. Once the subcutaneous (s.c.) tumor reached 1 to 1.5 cm in diameter, it was removed and cut into approximately 1- to 2-mm cubes and implanted in the left liver lobe of the mice. Two to four months after tumor implantation and appropriated treatments, the mice were sacrificed for examining the primary tumor growth on middle liver lobe and secondary tumor foci on left and right lobes. Nodules with diameters exceeding 0.1 to 0.2 cm observed on the left or right lobes were denoted as secondary tumor foci. Intrahepatic metastasis was defined if a minimum of two secondary tumor foci can be observed in the left and/or right liver lobes of a treated mouse. Extrahepatic metastasis was defined by tumors appearing in organs other than the liver, such as the intestine. During animal experiment, which was approved by the Institutional Animal Care and Use Committee at Tzu Chi University (No. 102080), regulations relevant to the care and use of laboratory animals were followed.

### Receptor array analysis

A receptor array kit (Proteome Profile™ Array; R&D system, Minneapolis MN, USA) capable of simultaneously detecting 49 essential RTKs in phosphorylated form was used for screening the phosphorylated RTK (p-RTK) signaling in HCC329 according to the manufacturer’s protocols. For quantitation, the average signal (pixel density) of the pair of duplicate spots representing each p-RTK was determined using Image J. The average background (from the PBS control spot) was then subtracted from each p-RTK signal. Detailed information of the 49 RTKs is presented in S2 Materials.

### Statistical analysis

A paired Student’s *t* test was conducted to analyze the differences in band intensities between samples on the Western blot and the differences in cell motility and doubling time between the indicated HCCs. Quantitative data were expressed as mean ± coefficient variation (CV), indicated by the error bars in each figure. Differences in intrahepatic metastasis between various treatments and the reproducibility of the indicated molecules exhibited on IHC tissue section were analyzed using the Fisher Exact V2 test for categorical data.

## Results

### Screening HGF/c-Met signaling in hepatocellular carcinomas

The status of HGF/c-Met signaling in 26 HCC tissue samples was evaluated by examining the amount of Tyr1234 phosphorylated c-Met (p-c-Met Tyr1234, the activated form of c-Met) coupled with HGF in the tumor environment. S1A Fig. depicts the IHC staining of p-c-Met and HGF on the HCC tissue sections from three HCC cases that were representative of HCCs with positive c-Met signaling. Prominent expressions of p-c-Met and HGF were detected within the tumor, but were absent in the non-tumor area of the tissue section (Fisher test, p<0.03, N = 3). The location of the HCC tumor was verified using the IHC of the HCC marker GPC3 coupled with hematoxylin/Eosin staining. Thus far, the proportion of the HCCs with active HGF/c-Met signaling was estimated to be 46% of the 26 HCC cases.

### Cellular phenotypes of patient-derived hepatocellular carcinoma

**Figure 1:**
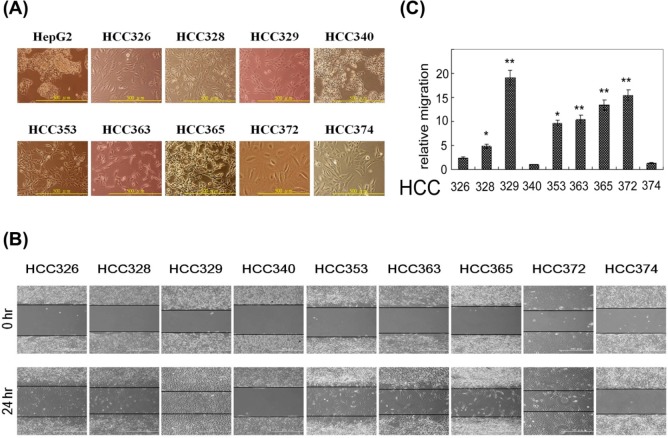
Comparison of cell morphologies and motilities among patients derived HCCs. The patient-derived HCC cells were established according to the description in Materials and Methods. Morphologies (A) and (B) motilities (by wound healing assay) of the indicated HCC cell lines were imaged using phase contrast microscopy (200X). (C) Quantitation for comparing the motility of the indicated HCCs. Relative motility of the HCCs was calculated; taking motility of HCC340 as 1.0. (**) and (*) represent statistical significance (p<0.005 and p<0.05, respectively, n = 4) for differences in motility between the indicated HCCs and the HCC340.

**Figure 2:**
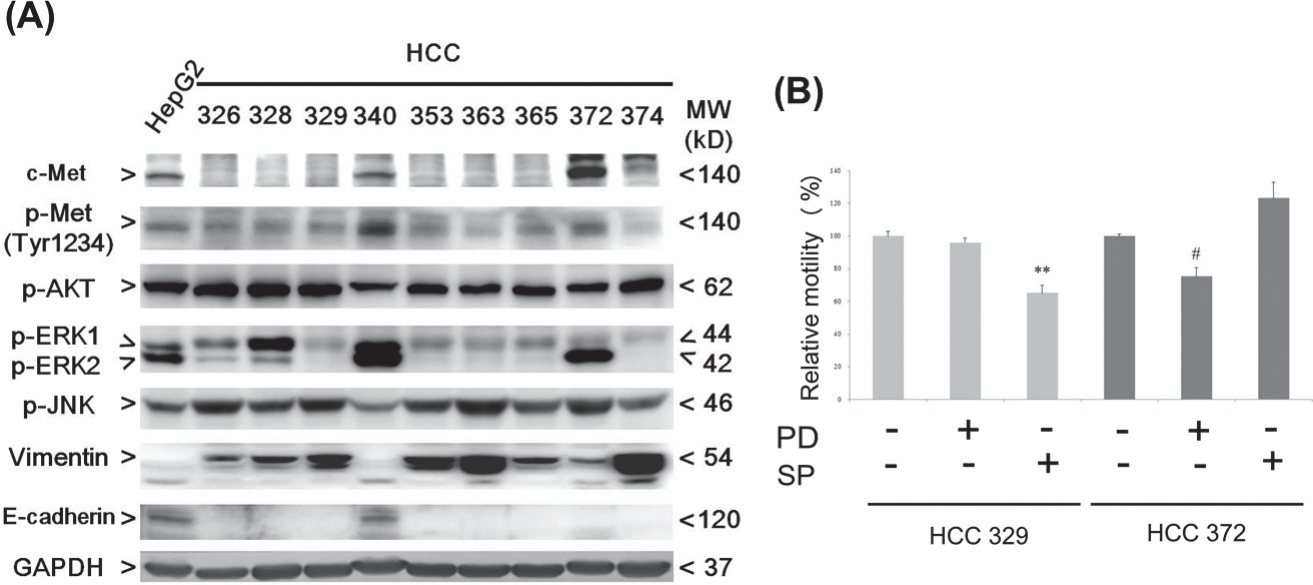
Comparison of various signaling molecules in HCC cell lines and suppression of cell migration by JNK and MEK inhibitors. (A) Western blots of indicated signal molecules in the indicated HCC cell lines using GAPDH as a loading control. Data were representative of two reproducible experiments. (B) Cells were untreated or treated with SP (SP600125), JNK inhibitor, or PD (PD98059), and subjected to wound healing migration analysis for 24 h. Relative migration was calculated and taking the data of untreated HCC372 or HCC329 as 100%. (**) and (#) represent statistical significance (p<0.005 and p<0.05, respectively, n = 3) between the indicated inhibitor-treated sample and untreated HCC329 or HCC372 control group.

To obtain more specific target cells for personalized therapy, parts of the HCC tissues from each of the 26 HCC cases were used for establishing patient-derived cell lines by feeder layer selection method. Currently, nine patient-derived HCC cell lines (HCC329, 328, 326, 340, 353, 365, 363, 372, and 374) were established and the phenotypes were characterized. HepG2, which is an epithelial and non-motile HCC, was also included for comparison of the HCC phenotypes. According to the morphologies demonstrated under phase contrast microscope (Fig. 1A), most of the HCCs (HCC326 328, 329, 353, 365, 363, 374) appeared mesenchymal, whereas only HCC340 and HepG2 appeared epithelial. The cellular size of HCC372 was larger than that of the other HCCs and its phenotypes was difficult to ascertain. To validate the phenotypes of the HCCs, Western blots of E-cadherin and vimentin, the typical epithelial and mesenchymal markers, respectively, were performed. As demonstrated in Fig. 2A, E-cadherin significantly expressed in HCC340 and HepG2, but not in the other HCCs. By contrast, vimentin expressed in all of the HCCs except HCC340 and HepG2. Thus, HCC340 and HepG2 can be classified as epithelial, and the other HCCs as mesenchymal. Migratory activity (assayed using the wound healing method for 24 h) of the patient-derived HCCs was determined (Fig. 1B) and quantitatively compared (Fig. 1C). As expected, the motility of HCC340 was lower than that of most mesenchymal HCCs (Fig. 1C). In addition, the motility of HCC329 and HCC372 was the highest among the nine HCCs.

### Analyzing the signaling status in patient-derived hepatocellular carcinomas

The statuses of the critical signaling components, including c-Met, ERK, JNK, and AKT, involved in tumor progression [47, 48] were examined in the patient-derived cell lines and HepG2. As shown in Fig. 2A, c-Met (β subunit with molecular weight of 140 kD) was highly expressed in HCC372, HCC 340 and HepG2, but was absent in the other cell lines. It was known that c-Met dimerization activates the phosphorylation of tyrosine residues (Tyr1234) in the kinase domain, leading to autophosphorylation of the carboxyl-terminal substrate-binding site at Tyr1349 and Tyr1356 [18]. Consistent with the profile of c-Met expression, p-c-Met (Tyr1234) was clearly detected in all c-Met positive HCCs, HCC372, HCC340, and HepG2, but was barely detected in the c-Met-negative HCCs (Fig. 2A). In addition, the other two Tyr-phosphorylated-c-Met (p-c-Met Tyr1356 and Tyr1349) were not detected in all cell lines (data not shown). In general, the status of p-c-Met in the patient-derived cell lines was the same as that observed in the corresponding primary tissues. As depicted in S1B Fig., the IHC of p-c-Met (Tyr1234) on the tissue section of HCC340 revealed dark staining, contrasting with the slight background staining observed on the parallel negative control section. This staining was consistent with the positive p-c-Met (Tyr1234) in the HCC340 cell line detected by Western blot (Fig. 2A). The IHC of p-c-Met (Tyr1234) for HCC372 (the other c-Met positive HCC) was not performed because tissue samples were lacking. The IHC of p-c-Met (Tyr1234) for other HCCs was negative (data not shown).

Regarding the downstream signaling components, p-JNK was abundant in most of the HCCs, and relatively low in HCC340 and HepG2 (Fig. 2A). On the other hand, the level of both phosphorylated ERK1 and ERK2 (p-ERK1 and p-ERK2, respectively) were markedly higher in HCC340 and relatively higher in HepG2. Also, p-ERK2 (but not p-ERK1) was abundant in HCC372, whereas p-ERK1 (but not p-ERK2) was very high in HCC328 and relatively high in HCC326 (Fig. 2A). In the other HCCs, including HCC329, HCC353, HCC363, HCC365, and HCC374, only p-ERK1 was marginally detected. In addition, abundant p-AKT was detected in most of the HCCs. In summary, although c-Met signaling was positive in only some of the cell lines (including HCC340, HCC372, and HepG2) and negative in others (including HCC326, 329, 353, 363, 365 and 374), the downstream ERK, JNK, and AKT were active in most of the HCCs. Thus, both c-Met dependent and independent signaling existed in the HCCs.

It appeared that the correlation of ERK and JNK activations with the status of c-Met signaling differed among the HCCs. As shown in Fig. 2A, p-ERK was higher in the c-Met-positive HCCs (including HCC340 and HCC372) than in most c-Met-negative HCCs (HCC329, 353, 363, 365, 372, and 374); whereas p-JNK was higher in most of the c-Met-negative HCCs than in the c-Met-positive HCCs (HCC340 and HepG2). However, some exceptions were observed. For example, p-ERK was high in the c-Met-negative HCC326 and HCC328, whereas p-JNK was high in the c-Met-positive HCC372. To further investigate the role of ERK and JNK in the tumor progression of c-Met-positive and c-Met-negative HCCs, we examined the effects that inhibitors of JNK (SP600125, SP) and MEK (the upstream kinase of ERK, PD98059, PD) exerted on cell migration of the two most motile HCCs, HCC372 and HCC329 (c-Met positive and c-Met negative HCC, respectively). As shown in Fig. 2B, SP, not PD, suppressed 35% of the migration (assayed by wound healing method for 24 h) of HCC329. By contrast, PD, not SP, suppressed 24% of the cell migration of HCC372. Thus we suggested that active ERK and JNK were required for mediating cell migration of c-Met-positive HCC and c-Met-negative HCC, respectively.

### LZ8 prevented migration and decreased cell survival of hepatocellular carcinoma

**Figure 3:**
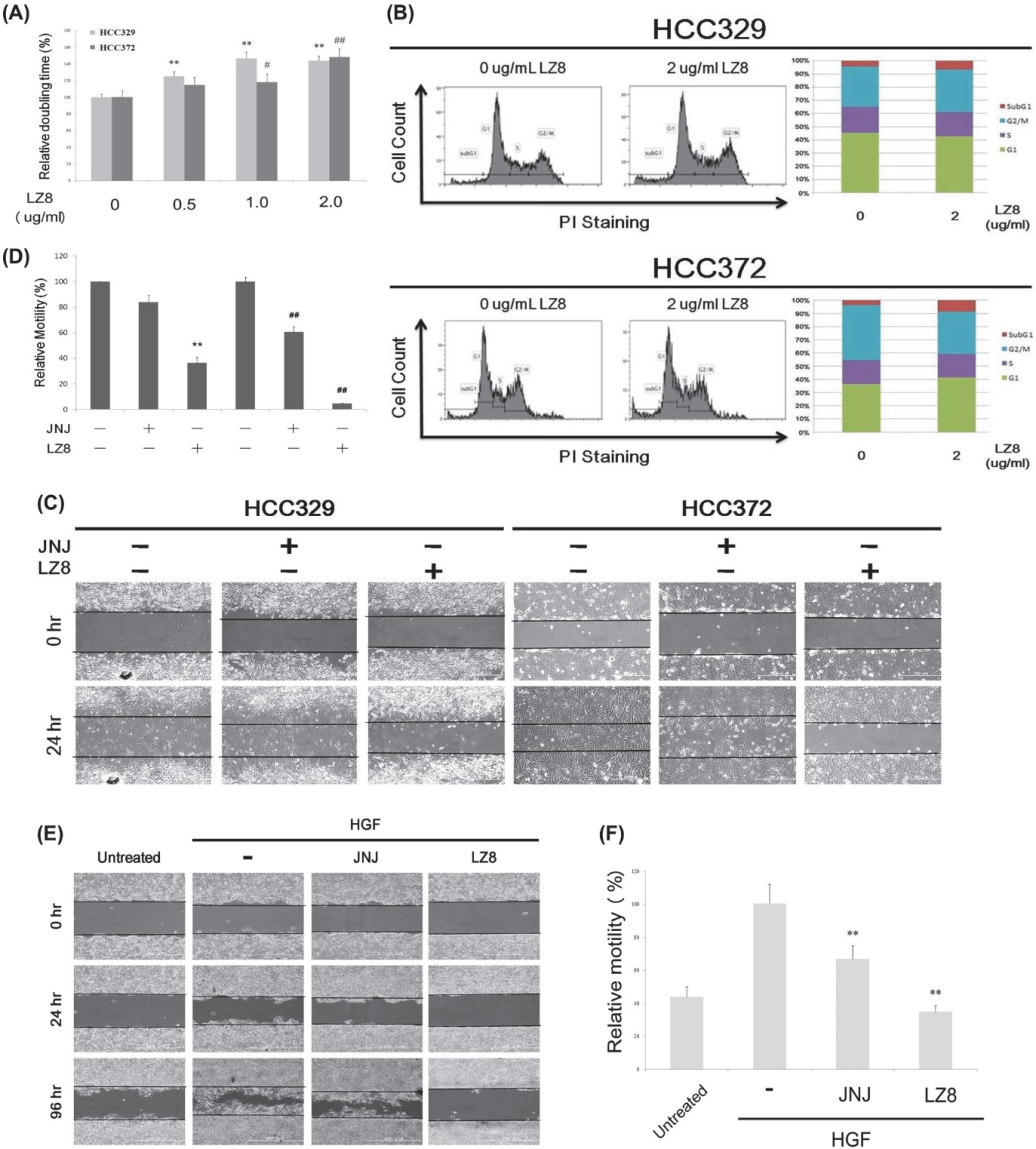
LZ8 prevented cell migration and reduced HCC survival. HCC329 and HCC372 cells were untreated (control) or treated with LZ8 (2.0 μg/mL) for 72 h (A and B), and LZ8 (2.0 μg/mL) or JNJ (26.5 nM) for 48 h (C). HepG2 cells were untreated (control), treated with 25 nM HGF with or without indicated inhibitors for 48 h (E). Doubling time determination (A) and cell cycle analysis (B) at 72 h, and wound healing migration analysis (C) and (E) of the HCCs were performed. In (A), relative doubling time was evaluated taking the data of untreated HCC372 or HCC329 as 100%. In (B), the cell counts of each cell cycle phase are depicted in the left panel (PI: propidium iodide), whereas the percentage of each phase is shown in the right panel. Data were the average of three experiments; CV = 5.8%. (D) and (F) are quantitative figures for (C) and (E), respectively. In (A) and (D), (**) and (*) (#) represent statistical significance (p<0.005 and p<0.05, respectively, n = 3) between the indicated inhibitor-treated samples and the untreated HCC329 or untreated HCC372 group. In (F), (**) represent statistical significance (p<0.005, n = 3) between the indicated HGF/inhibitor samples and the HGF-only group.

Both HCC372 and HCC329 were further used as target cells for evaluating the potential of LZ8 as an anti-HCC agent. As shown in the cell proliferation assay (Fig. 3A), LZ8 (0.5, 1.0, and 2.0 μg/mL) dose dependently increased the doubling times of HCC329 and HCC372 by 25% to 46% and 14% to 47%, respectively. To investigate the underlying mechanisms of the antiproliferative effect of LZ8, cell cycle analysis was performed. As demonstrated in Fig. 3B, treating HCC372 and HCC329 with LZ8 (2.0 μg/mL) for 72 h did not delay cell cycle progression, because the percentages of the G1 and S phase of LZ8-treated cells were not significantly different from those of the untreated cells. However, LZ8 treatment increased the sub-G1 proportions of HCC372 and HCC329 by 5% to 10% compared with those of the untreated HCCs, suggesting that LZ8 may decrease the cell survival of a small population of both HCCs.

Further, the effect that LZ8 exerted on the cell migration of both HCCs was examined using the wound healing assay. As demonstrated in Fig. 3C and 3D, LZ8 treatment (2.0 μg/mL) for 24 h markedly suppressed cell migration of HCC372 by 95%. Also, the c-Met specific antagonists JNJ-38877605 (JNJ, at IC_50_: 26.5 nM) suppressed migration of HCC372 by 50%. On the other hand, LZ8 and JNJ suppressed cell migration of HCC329 by 80% and 10%, respectively. Notably, the extent of inhibition by JNJ was higher in HCC372 than in HCC329, a phenomenon that can be attributed to c-Met signaling which was active in HCC372 but not HCC329. In addition, compared with the single LZ8 treatment, a combination of both LZ8 and JNJ didn’t exert enhanced effects in blocking the migration of both HCCs (data not shown). Because the cells were treated with LZ8 in serum-free media in the wound healing assay, whether serum can influence the effect that LZ8 exerted on HCC migration needed to be examined. Thus, we repeated the cell migration assay by using a transwell culture insert, and treated HCC329 and HCC372 with LZ8 in complete media containing 10% serum. As depicted in S2 Fig., LZ8 (2.0 μg/mL and 5.0 μg/mL) substantially suppressed the transwell migration of HCC372 and HCC329 in a dose-dependent manner. Thus, the preventive effects that LZ8 exerted on HCC cell migration were unaffected by serum. In summary, LZ8 can markedly suppress the constitutive cell migration of both c-Met-positive and c-Met-negative HCC. In addition, LZ8 (2.0 μg/mL) prevented the HGF-induced cell migration of HepG2 much more effectively than did JNJ (26.5 nM) (Fig. 3E and 3F).

### LZ8 markedly suppressed tumor progression of HCC329 in SCID mice

**Figure 4:**
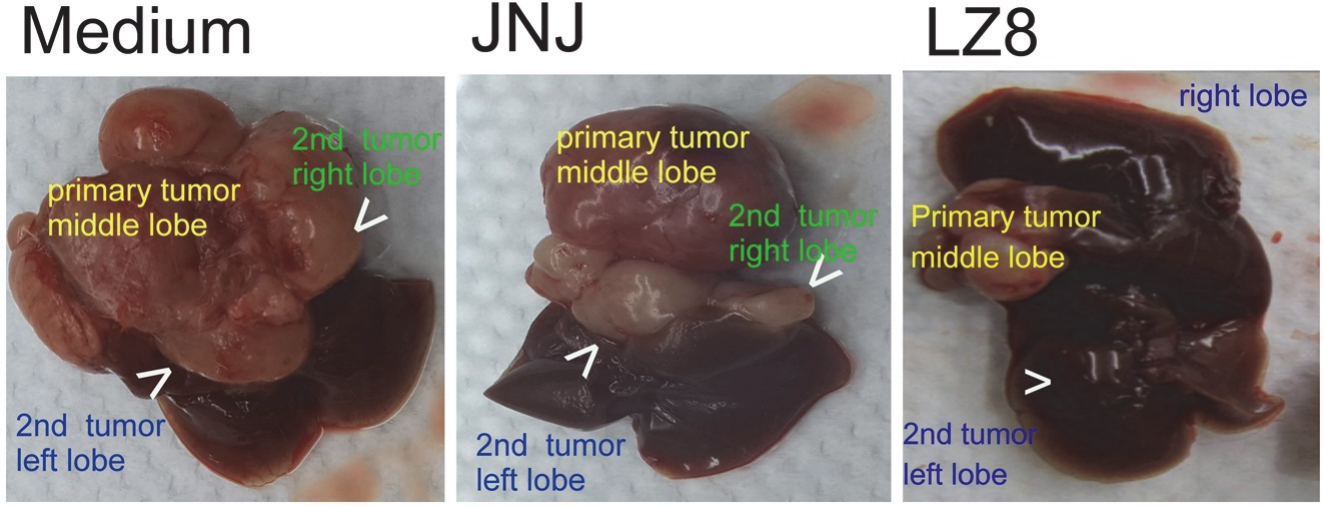
LZ8, not JNJ, prevents tumor metastasis of HCC329. HCC329 was inoculated onto the middle lobe of SCID mouse livers through orthotropic transplantation. Subsequently, the mice were injected intraperitoneally twice per week with DMEM medium, JNJ-38877605 (JNJ) 26.5 nmole/g per mouse, or LZ8 (4.0–20.0 μg/g per mouse). The mice were sacrificed 2 months after injection; intrahepatic metastasis was observed in the medium- and JNJ- but not LZ8-treated group. White arrow heads indicate the site of secondary tumors on the left and right liver lobes. Data were representative of two reproducible experiments.

We further examined the effect that LZ8 exerted on HCC329 metastasis using a SCID mouse model established previously [45]. HCC329 was orthotropically transplanted into the middle liver lobe of SCID mice, and then DMEM (vehicle), vehicle-containing LZ8 (20 μg/g mouse), or JNJ (2.0 nmole/g mouse) were injected intraperitoneally. Interestingly, LZ8 radically suppressed HCC329 tumor progression in the SCID mice. As demonstrated in Fig. 4, primary tumor growth on the middle liver lobe and intra-metastasis toward the right and left liver lobes in the LZ8-treated mice markedly reduced, compared with those in the DMEM- and JNJ-treated mice (Fisher test: p<0.05, N = 3). The toxicity test for LZ8 was performed by treating four normal SCID mice with high dose LZ8, without prior inoculation of HCC329 on the liver. After 2 to 3 months of intraperitoneal LZ8 administration (20 μg/g mouse), no adverse effects regarding the activity of the mice and the integrity of organs were observed (data not shown).

### Effect of LZ8 on c-Met-dependent and c-Met-independent signaling in hepatocellular carcinoma

**Figure 5:**
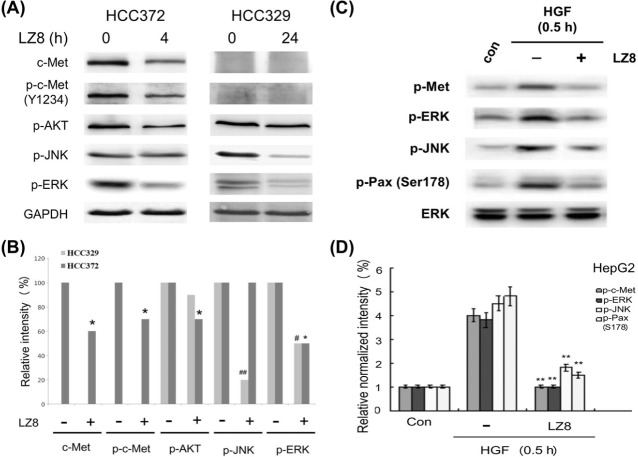
LZ8 block signal transduction in c-Met positive and negative HCCs and HGF-induced HepG2. HCC372 and HCC329 were treated with 2.0 μg/mL LZ8 for 4 h and 24 h, respectively (A), and HepG2 was untreated (control), treated with HGF only, or treated with HGF coupled with 2.0 μg/mL of LZ8 (C). Western blotting of indicated signal molecules was performed using GAPDH (A) and ERK (C) as loading controls. (B) and (D) are quantitative figures for (A) and (C), respectively. Relative band intensity of each molecule was calculated; taking the data of untreated HCC372 and HCC329 as 100% (B) and untreated HepG2 as 1.0 (D). In (B), (**) (^##^) and (*) (^#^) represent statistical significance (p<0.005 and p<0.05, respectively, n = 3) between the indicated inhibitor-treated sample and untreated HCC329 or HCC372 control group. In (D), (**) represent statistical significance (p<0.005, n = 3) between the indicated HGF/LZ8 cotreated and the HGF-only group.

To investigate the molecular mechanisms by which LZ8 suppresses HCC progression, we examined the influence that LZ8 exerts on the activity of critical signal molecules in HCC329 and HCC372. As shown in Fig. 5A and 5C, the phosphorylation of JNK, ERK, and AKT, decreased, by 85%, 51%, and 18%, respectively, after treatment of HCC329 with LZ8 (2.0 μg/mL) for 24 h. The effect that LZ8 exerted on the signal transduction in HCC372 differed from that in HCC329 in several aspects. First, the suppressive effect of LZ8 was observed earlier in HCC372 (at 3–4 h) than in HCC329 (at 24 h). Second, LZ8 altered c-Met-related signal molecules in the c-Met-positive HCC372, but not in c-Met negative HCC329. As shown in Fig. 5A and 5C, expression of c-Met was greatly suppressed by LZ8 by 90% to 95% at 4 h in HCC372, which may sustain until 24 h (data not shown). Consistently, LZ8 significantly decreased p-c-Met (Tyr1234) by 55% at 4 h (Fig. 5A and 5C). On the downstream level, LZ8 abolished p-ERK and p-AKT by 80% to 95% at 4 h (Fig. 5A and 5C). However, LZ8 did not reduce p-JNK in HCC372. In summary, LZ8 suppressed activities of critical signal molecules in both c-Met-positive and c-Met-negative HCCs.

### Screening the active RTK signaling that can be blocked by LZ8 in HCC329

**Figure 6:**
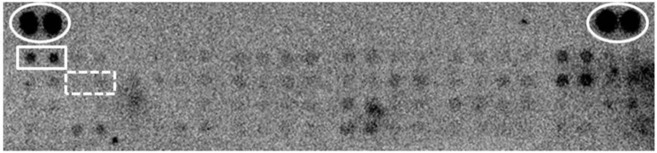
Receptor array analysis for detecting RTK signaling in HCC329. Total cell lysate (300 μg/mL) of untreated HCC329 was used for analyzing the relative amounts of 49 phosphorylated RTKs by receptor array analysis, as described in Materials and Methods. The rectangles depicted by solid and dashed lines indicate the duplicated positions of p-EGFR and p-c-Met, respectively. The elliptic circles indicate the position of two positive controls.

Because c-Met signaling is inactive in HCC329, the signaling pathway suppressed by LZ8 for blocking tumor progression of this HCC remained unclear. Thus, the RTK signaling activated in HCC329 was screened using a receptor array, which can simultaneously detect 49 essential RTKs in phosphorylated forms. Among the phosphorylated RTKs, phosphorylated EGFR (p-EGFR) was the most abundant in untreated HCC329 (Fig. 6). Moreover, p-c-Met (p-HGFR) was undetectable in the same array of untreated HCC329, consistent with the negative p-c-Met (Tyr1234) determined using the Western blot (Fig. 2A). Furthermore, p-EGFR was markedly reduced in HCC329 treated with LZ8 for 16 h, compared with that in untreated HCC329 (data not shown). Table 1 shows the quantitative changes of ten representative p-RTKs, including p-EGFR, after LZ8 treatment compared with those in the untreated HCC329. Notably, the relative intensity of p-EGFR in LZ8-treated HCC329 *vs*. that in untreated HCC329 (LZ8/control) was 0.317, far below 1.0. This revealed that p-EGFR was greatly suppressed in HCC329 after LZ8 treatment. In addition, the relative intensities of p-Axl and p-RYK were significantly less than 1.0, whereas those of the other seven p-RTKs were greater than 1.0. On the other hand, we investigated whether blocking the EFGR pathway would affect the signal transduction and cell migration of HCC329. As depicted in S3A Fig., inhibitors of EGFR, AG1748, and SU5416 suppressed the cell migration (using wound healing method) of HCC329 by 80%. Moreover, treating HCC329 with AG1748 reduced the level of p-JNK by 30% within 4 to 16 h (S3B Fig.). In summary, EGFR-JNK signaling was required for mediating cell migration of HCC329, which can be effectively suppressed by LZ8.

**Table 1:** Comparison of intensities (on receptor array) of p-RTKs in LZ8-treated HCC329 *vs*. that in untreated HCC329.

**Receptor**	**Intensity^a^**		**Relative Intensity^b^**
	**Control**	**LZ8**	**LZ8/Control**
**EGF R**	0.104	0.033	**0.317**
**FGF R4**	0.036	0.063	1.750
**Axl**	0.135	0.074	**0.548**
**PDGF Rα**	0.053	0.063	1.189
**c-Ret**	0.070	0.091	1.300
**ROR1**	0.014	0.021	1.500
**TrkC**	0.056	0.069	1.232
**EphA3**	0.061	0.066	1.082
**ALK**	0.065	0.079	1.215
**RYK**	0.129	0.098	**0.760**

^a^ Intensities of the indicated phosphorylated RTKs were calculated as described in Materials and Methods.

^b^ LZ8/control represents the ratios of intensities of the indicated p-RTK signals on the array for LZ8-treated HCC329 versus that for the untreated group (control). Data were the averages of two reproducible experiments. The number in bold type indicates the relative intensity (LZ8/control) of p-RTK that was less than 1.0.

Finally, we investigated whether LZ8 could block HGF-induced c-Met signaling in HepG2. As shown in Fig. 5B, LZ8 suppressed the HGF-induced c-Met signaling [45], including p-c-Met, p-JNK, p-ERK, and p-paxilllin (Ser 178) by 85% to 90% in HepG2 (Fig. 5D). Moreover, LZ8 suppressed the HGF-induced cell migration of HepG2 (assayed by wound healing method at 24 h and 96 h) more efficiently than did JNJ.

## Discussion

### Screening of c-Met-positive hepatocellular carcinoma for c-Met-based target therapy

Target therapies are emerging as the most promising strategy for HCC management. Among the growth-factor-mediated cancer signaling, the HGF/c-Met axis is intimately related to the metastatic transformation of HCC. A prerequisite of c-Met-based target therapy is to identify whether c-Met signaling is active in an individual HCC, an aspect that has not been clarified in previous clinical studies. In our initial screening, p-c-Met (Tyr1234), the active form of c-Met, was positive in 46% of the HCCs tested. Although the sample size was not large enough, this pilot study suggests that large-scale screening of c-Met signaling in HCC is feasible before enrolling patients for c-Met-based target therapy.

### Establishing and characterizing patient-derived hepatocellular carcinoma

Another critical concern in the preclinical trials for anti-HCC progression is selecting target cells for studying the efficacy of various HCC antagonists. In this study, numerous patient-derived HCC cells were established and characterized (Fig. 1). After the expression and phosphorylation of c-Met in HCC cell lines was screened, it appeared that approximately 33% of the HCCs were positive of c-Met signaling (Fig. 2A). Moreover, the status of p-c-Met in HCC cell line recapitulates those observed in the corresponding primary tissues, indicating that the status of c-Met signaling in the original HCC tissue can be sustained in the cell lines.

### Status of c-Met signaling and the epithelial/mesenchymal phenotype of hepatocellular carcinoma

The status of c-Met signaling in the HCCs was essential for verifying whether patients are suitable for c-Met-based target therapy. This was reflected by that the suppressive effect of JNJ, the typical c-Met inhibitor, exerted on cell migration was much more prominent for HCC372 than for HCC329 (Fig. 3C and 3D). However, whether c-Met signaling was essential for maintaining the mesenchymal phenotype and motility of HCC remained uncertain. According to the profile of E-cadherin and vimentin (Fig. 2A), HCC340 and HepG2 can be classified as epithelial, whereas the other HCCs are mesenchymal type. On other hand, c-Met and p-c-Met were observed in HCC372, HCC340, and HepG2, but not in other HCCs (Fig. 2A). In summary, active c-Met signaling was observed in two epithelial HCCs (HCC340 and HepG2) and only one mesenchymal HCC (HCC372), but not evident in most other mesenchymal HCCs. Therefore, positive c-Met signaling was weakly correlated with the mesenchymal phenotype of the HCCs. This finding contradicts the notion that HGF/c-Met signaling is the essential pathway responsible for triggering EMT and cell migration that leads to tumor metastasis (2, 27–29). A possible explanation is that although HGF/c-Met signaling is required for triggering EMT of HCC, c-Met signaling may be down regulated after the EMT process is completed and compensated for by other RTK pathways for sustaining the mesenchymal phenotype and high motility. This notion can be supported by the fact that EGFR, but not c-Met signaling, is active in HCC329 (Fig. 6), which is a typically mesenchymal and highly motile HCC (Figs. 1B, 1C, and 2A).

### Effect of LZ8 on tumor progression of hepatocellular carcinoma

Effective and safe drugs are required for successful HCC target therapy. Although the usefulness of sorafenib has been demonstrated, developing second line drugs for HCC patients who were resistant to sorafenib are essential. Currently, many potential Chinese-herb-derived anticancer compounds have been explored. For example, Celastrol, extracted from the root of Tripterygiumwilfordii Hook, was found to have anti–prostate cancer properties [51]. Moreover, Platycodin D (PD) [52] and TDP [53], isolated from the traditional Chinese herb, Platycodonis radix and Garcinia oblongifolia, respectively, have anticancer potential in HCC. In this study, we determined that LZ-8 prevents the tumor progression of patient-derived HCC in vitro (including cell migration and cell survival) (Fig. 3) and intrahepatic metastasis of HCC329 in SCID mice (Fig. 4). The suppressive effect of LZ8 exerted on cell migration (Fig. 3C and 3D) was much higher than that on decreasing cell survival (Fig. 3B), possibly because the signal pathways affected by LZ8 were primarily responsible for cell migration of HCC.

### LZ8: A potent antagonist of c-Met-dependent and c-Met-independent signaling

The effect that LZ8 exerted on HCC tumor progression can be ascribed to the suppression of critical signaling, either c-Met dependent or c-Met independent, involved in HCC progression. For the c-Met positive HCC372, LZ8 suppressed both the expression of c-Met and phosphorylation of c-Met (Tyr1234) (Fig. 5A), which accompanied the decrease of the downstream ERK signaling (Fig. 5A) and the inhibition of cell migration (Fig. 3C and 3D). This is consistent with previous studies demonstrating that knockdown of c-Met by antisense RNA or RNA interference significantly inhibited the growth of HCC cells with high levels of c-Met expression [54–56]. The underlying mechanism by which LZ8 suppresses the expression of c-Met remains unclear. One possibility is that LZ8 may inhibit transcription of c-Met. Alternatively, LZ8 may promote the degradation of c-Met associated with c-Met endocytosis [45]. In addition, the suppressive effect of LZ8 exerts on HCC migration was much stronger than that of the conventional c-Met inhibitor JNJ (Fig. 3C and 3D). This was consistent with the observation that LZ8 suppressed c-Met expression much more than JNJ (S4 Fig.).

According to receptor array screening, we determined that EGFR was the major RTK activated in the c-Met-negative HCC329 (Fig. 6). Moreover, p-EGFR can be suppressed by LZ8, although the underlying mechanism remained unclear. Consistently, cell migration of HCC329 can be prevented by the EGFR inhibitor AG1748 (S3A Fig.). Taken together, LZ8 may suppress EGFR signaling, thereby suppressing tumor progression of HCC329. In the future, the LZ8-sensitive signaling pathway in other c-Met-negative HCCs can also be identified using the receptor screening approach.

### MAPK cascade in the patient-derived hepatocellular carcinoma

We established the profile of downstream MAPK members, including p-JNK and p-ERK, in the patient-derived HCCs. Because of their essential roles in proliferation, survival, differentiation, and migration, deregulated MAPKs were frequently found to contribute to the development of many cancers, including HCC [47]. Previously, the role of p-ERK in the growth and survive of HCC has already been established [2]. Recently, the role of JNK in HCC progression is also emerging. Inhibiting JNK1 expression can reduce the migration and invasion of mouse HCC cell lines *in vitro* [49]. In the present study, differential degrees of JNK and ERK activation were observed in both c-Met-positive and c-Met-negative HCCs. Notably, active ERK was required for mediating the cell migration of c-Met-positive HCC372 (in which ERK2 activation is high), whereas active JNK was required for the cell migration of c-Met-negative HCC329 (in which JNK activation is high) (Fig. 2B). Moreover, ERK2 phosphorylation was dramatically high in the c-Met-positive HCC, whereas ERK1 phosphorylation was marginally detected in most of the c-Met-negative HCCs (Fig. 2A). This is reminiscent of a previous study suggesting that ERK2, but not ERK1, mediated the HGF-induced motility of non–small cell lung carcinoma cell lines [50]. Therefore ERK2 may be more essential than ERK1 for the tumor progression of HCC triggered by HGF.

The suppressive effects of LZ8 on MAPK in both HCC329 and HCC372 also differed substantially. Although phosphorylation of ERK and JNK can be detected in HCC372 (Fig. 2A), only p-ERK (but not p-JNK), was suppressed by LZ8 (Fig. 5A). One possible explanation is that JNK was not activated via the c-Met-dependent pathway in HCC372, and therefore not influenced by LZ8. On the contrary, LZ8 suppressed p-JNK much more than p-ERK in HCC329, a phenomenon that is consistent with the suppression of p-EGFR by LZ8 (Table 1). In addition, the phosphorylation of JNK can be markedly suppressed by the EGFR inhibitor AG1478 (S3B Fig.). These results strongly suggested that JNK is downstream of EGFR in HCC329, and the EGFR-JNK axis is a promising target for LZ8 to suppress the tumor progression of this HCC.

Recently, the roles of p-JNK and p-ERK as prognosis markers of HCC treated with sorafenib have been studied. Compared with ERK activation, JNK activation is more inversely correlated with the therapeutic response to sorafenib, suggesting that JNK activity may be used as a predictive biomarker for response to sorafenib treatment [57–59]. In this regard, whether p-JNK and p-ERK can be used as prognosis markers for LZ8-treated HCC warrants further investigation.

## Conclusion

To develop an effective c-Met-based target therapy for preventing HCC progression, we screened c-Met-dependent signaling on HCC tissues. In addition, patient-derived cell lines, either c-Met-positive or c-Met-negative, may be used for testing the efficacy of HCC antagonists in vitro and in vivo. Specifically, LZ8 suppressed the tumor progression of HCC *via* blocking the c-Met-dependent or the c-Met-independent pathway. Since the safety concern of LZ8 has been certified, whether it can be used as an effective and nontoxic anti-HCC agent is worthy of further investigation in clinical trials.

## Supporting Information

S1 Fig Immunohistochemistry of p-c-Met and HGF in HCC tumors. (A) IHC of the indicated molecules coupled with H & E stain was performed on tissue sections of three HCC cases. Green arrows indicate the location of tumor as verified by HCC tumor marker GPC3. The blue rectangle indicates the area that was magnified to 400×. The deep brown staining region revealed the location of indicated molecules in contrast to the light brown negative region. Data were representative of two reproducible experiments. (B) IHC of p-c-Met coupled with H & E stain was performed on tissues from which the indicated HCCs cell lines were derived. Imaging was performed through phase contrast microscopy. The image of negative controls (excluding the primary Ab incubation) was demonstrated in parallel. The area indicated by blue rectangles (100X magnification) was enlarged to 400X magnification. In the IHC of p-c-Met, the deep brown staining region revealed the location of p-c-Met in contrast to the light brown negative region. Data were representative of two reproducible experiments.
(TIF)

S2 Fig LZ8 prevented transwell migration of HCC329 and HCC372 in complete medium. HCC372 and HCC329 were cultivated on a migration culture insert for 24 h and treated with LZ8 at indicated concentrations in a medium containing 10% serum for 24 h. Imaging was performed through phase contrast microscopy using 200× magnification. Data were representative of three reproducible experiments.
(TIF)

S3 Fig EGFR inhibitors prevent JNK phosphorylation and cell migration of HCC329. HCC329 cells were untreated (control) or treated with indicated inhibitors for 48 h (A) or indicated times (B). Wound healing migration analysis (A) and Western blot analysis of p-JNK (B) were performed. In (A), relative migration time was calculated, taking the data of control group as 100%. In (B), GAPDH was included as the loading control. The normalized intensity calculated as p-JNK/GAPDH is shown. Data were representative of three reproducible experiments.
(TIF)

S4 Fig LZ8 suppressed c-Met expression more effectively than JNJ. HCC372 cells were treated with JNJ (26.5 nM) or 2 μg/mL LZ8 for 4 h. Western blot of indicated signal molecules was performed using GAPDH as a loading control. Data were representative of three reproducible experiments.
(TIF)

S1 Materials Patient signed informed consent forms for HCC cell line establishment. Signed informed consent forms (with partially hidden informations) of 4 HCC patients who agreed to provide their surgical HCC tissues for cell lines establishment in Liver Disease center in TZU CHI hospital Hualein, Taiwan.
(PDF)

S2 Materials Detailed information of the 49 RTKs in receptor array. This is one of the appendixes in the manufacture’s protocol of Proteome Profile™ Array; R&D system. It provides the detailed information regarding the positions of the antibodies of 49 phosphorylated RTKs (p-RTKs) conjugated on the membrane for detecting the respective p-RTKs. The letters indicated in the most left (1^st^) and 4^th^ column are the coordinates referring to the position of each antibody (in duplicate) indicated in the parallel column.
(JPG)

S3 Materials English editing certificate. The copied certificate of English editing for this paper by a mother tongue English speaker, Dr. Jennifer Sampson in Wallace Academic Editing Company.
(PDF)

## References

[1] Ferlay J, Shin HR, Bray F, Forman D, Mathers C, et al. (2008) Estimates of worldwide burden of cancer in 2008: GLOBOCAN 2008. Int J Cancer 127: 2893–2917. 10.1002/ijc.25516 21351269

[2] Gao J, Inagaki Y, Song P, Qu X, Kokudo N, et al. (2012) Targeting c-Met as a promising strategy for the treatment of hepatocellular carcinoma. Pharmacol Res 65: 23–30. 10.1016/j.phrs.2011.11.011 22138044

[3] Farazi PA, DePinho RA (2006) Hepatocellular carcinoma pathogenesis: from genes to environment. Nat Rev Cancer 6: 674–687. 10.1038/nrc1934 16929323

[4] Poon D, Anderson BO, Chen LT, Tanaka K, Lau WY, et al. (2009) Management of hepatocellular carcinoma in Asia: consensus statement from the Asian Oncology Summit 2009. Lancet Oncol 10: 1111–1118. 10.1016/S1470-2045(09)70241-4 19880065

[5] Bruix J, Sherman M (2005) Management of hepatocellular carcinoma. Hepatology 42: 1208–1236. 10.1002/hep.20933 16250051

[6] Llovet JM, Ricci S, Mazzaferro V, Hilgard P, Gane E, et al. (2008) Sorafenib in advanced hepatocellular carcinoma. N Engl J Med 359: 378–390. 10.1056/NEJMoa0708857 18650514

[7] Cheng AL, Kang YK, Chen Z, Tsao CJ, Qin S, et al. (2009) Efficacy and safety of sorafenib in patients in the Asia-Pacific region with advanced hepatocellular carcinoma: a phase III randomised, double-blind, placebo-controlled trial. Lancet Oncol 10: 25–34. 10.1016/S1470-2045(08)70285-7 19095497

[8] Llovet JM, Ducreux M, Lencioni R, Di Bisceglie AM, Galle PR, et al. (2012) EASL-EORTC clinical practice guidelines: management of hepatocellular carcinoma. J Hepatol 56: 908–943. 10.1016/j.jhep.2011.12.001 22424438

[9] Zhu AX (2012) Molecularly targeted therapy for advanced hepatocellular carcinoma in 2012: current status and future perspectives. Semin Oncol 39: 493–502. 10.1053/j.seminoncol.2012.05.014 22846866

[10] Zhai B, Sun XY (2013) Mechanisms of resistance to sorafenib and the corresponding strategies in hepatocellular carcinoma. World J Hepatol 5: 345–352. 10.4254/wjh.v5.i7.345 23898367 PMC3724962

[11] Bosch FX, Ribes J, Diaz M, Cleries R (2004) Primary liver cancer: worldwide incidence and trends. Gastroenterology 127: S5–S16. 10.1053/j.gastro.2004.07.015 15508102

[12] Tang ZY, Ye SL, Liu YK, Qin LX, Sun HC, et al. (2004) A decade’s studies on metastasis of hepatocellular carcinoma. J Cancer Res Clin Oncol 130: 187–196. 10.1007/s00432-003-0511-1 14685850 PMC12161827

[13] Gupta GP, Massague J (2006) Cancer metastasis: building a framework. Cell 127: 679–695. 10.1016/j.cell.2006.11.001 17110329

[14] Liotta LA, Kohn EC (2001) The microenvironment of the tumour-host interface. Nature 411: 375–379. 10.1038/35077241 11357145

[15] Christofori G (2006) New signals from the invasive front. Nature 441: 444–450. 10.1038/nature04872 16724056

[16] Gao CF, Vande Woude GF (2005) HGF/SF-Met signaling in tumor progression. Cell Res 15: 49–51. 10.1038/sj.cr.7290264 15686627

[17] Buijs JT, Stayrook KR, Guise TA (2011) TGF-beta in the Bone Microenvironment: Role in Breast Cancer Metastases. Cancer Microenviron 4: 261–281. 10.1007/s12307-011-0075-6 21748439 PMC3234330

[18] Gherardi E, Birchmeier W, Birchmeier C, Vande Woude G (2012) Targeting MET in cancer: rationale and progress. Nat Rev Cancer 12: 89–103. 10.1038/nrc3205 22270953

[19] Ueki T, Fujimoto J, Suzuki T, Yamamoto H, Okamoto E (1997) Expression of hepatocyte growth factor and its receptor c-met proto-oncogene in hepatocellular carcinoma. Hepatology 25: 862–866. 10.1002/hep.510250413 9096589

[20] Osada S, Kanematsu M, Imai H, Goshima S (2008) Clinical significance of serum HGF and c-Met expression in tumor tissue for evaluation of properties and treatment of hepatocellular carcinoma. Hepatogastroenterology 55: 544–549. 18613405

[21] Ke AW, Shi GM, Zhou J, Wu FZ, Ding ZB, et al. (2009) Role of overexpression of CD151 and/or c-Met in predicting prognosis of hepatocellular carcinoma. Hepatology 49: 491–503. 10.1002/hep.22639 19065669

[22] Wang ZL, Liang P, Dong BW, Yu XL, Yu de J (2008) Prognostic factors and recurrence of small hepatocellular carcinoma after hepatic resection or microwave ablation: a retrospective study. J Gastrointest Surg 12: 327–337. 10.1007/s11605-007-0310-0 17943391

[23] Kaposi-Novak P (2009) Comparative genomic classification of human hepatocellular carcinoma. Magy Onkol 53: 61–67. 10.1556/MOnkol.53.2009.1.9 19318328

[24] Ogunwobi OO, Liu C (2011) Hepatocyte growth factor upregulation promotes carcinogenesis and epithelial-mesenchymal transition in hepatocellular carcinoma via Akt and COX-2 pathways. Clin Exp Metastasis 28: 721–731. 10.1007/s10585-011-9404-x 21744257 PMC3732749

[25] Jiang Y, Xu W, Lu J, He F, Yang X (2001) Invasiveness of hepatocellular carcinoma cell lines: contribution of hepatocyte growth factor, c-met, and transcription factor Ets-1. Biochem Biophys Res Commun 286: 1123–1130. 10.1006/bbrc.2001.5521 11527416

[26] Ogunwobi OO, Wang T, Zhang L, Liu C (2012) Cyclooxygenase-2 and Akt mediate multiple growth-factor-induced epithelial-mesenchymal transition in human hepatocellular carcinoma. J Gastroenterol Hepatol 27: 566–578. 10.1111/j.1440-1746.2011.06980.x 22097969 PMC3288221

[27] Goyal L, Muzumdar MD, Zhu AX (2013) Targeting the HGF/c-MET pathway in hepatocellular carcinoma. Clin Cancer Res 19: 2310–2318. 10.1158/1078-0432.CCR-12-2791 23388504 PMC4583193

[28] You H, Ding W, Dang H, Jiang Y, Rountree CB (2011) c-Met represents a potential therapeutic target for personalized treatment in hepatocellular carcinoma. Hepatology 54: 879–889. 10.1002/hep.24450 21618573 PMC3181384

[29] Chan SL, Yeo W (2012) Targeted therapy of hepatocellular carcinoma: present and future. J Gastroenterol Hepatol 27: 862–872. 10.1111/j.1440-1746.2012.07096.x 22369685

[30] Pelicci G, Giordano S, Zhen Z, Salcini AE, Lanfrancone L, et al. (1995) The motogenic and mitogenic responses to HGF are amplified by the Shc adaptor protein. Oncogene 10: 1631–1638. 7731718

[31] Ponzetto C, Bardelli A, Zhen Z, Maina F, dalla Zonca P, et al. (1994) A multifunctional docking site mediates signaling and transformation by the hepatocyte growth factor/scatter factor receptor family. Cell 77: 261–271. 10.1016/0092-8674(94)90318-2 7513258

[32] Trusolino L, Bertotti A, Comoglio PM (2010) MET signalling: principles and functions in development, organ regeneration and cancer. Nat Rev Mol Cell Biol 11: 834–848. 10.1038/nrm3012 21102609

[33] Zhu K, Kong X, Zhao D, Liang Z, Luo C (2014) c-MET kinase inhibitors: a patent review (2011–2013). Expert Opin Ther Pat 24: 217–230. 10.1517/13543776.2014.864279 24266843

[34] Diaz D, Ford KA, Hartley DP, Harstad EB, Cain GR, et al. (2013) Pharmacokinetic drivers of toxicity for basic molecules: strategy to lower pKa results in decreased tissue exposure and toxicity for a small molecule Met inhibitor. Toxicol Appl Pharmacol 266: 86–94. 10.1016/j.taap.2012.10.026 23142475

[35] Santoro A, Simonelli M, Rodriguez-Lope C, Zucali P, Camacho LH, et al. (2013) A Phase-1b study of tivantinib (ARQ 197) in adult patients with hepatocellular carcinoma and cirrhosis. Br J Cancer 108: 21–24. 10.1038/bjc.2012.556 23287988 PMC3553536

[36] Xue Q, Ding Y, Shang C, Jiang C, Zhao M (2008) Functional expression of LZ-8, a fungal immunomodulatory protein from Ganoderma lucidium in Pichia pastoris. J Gen Appl Microbiol 54: 393–398. 10.2323/jgam.54.393 19164882

[37] Lin YL, Liang YC, Tseng YS, Huang HY, Chou SY, et al. (2009) An immunomodulatory protein, Ling Zhi-8, induced activation and maturation of human monocyte-derived dendritic cells by the NF-kappaB and MAPK pathways. J Leukoc Biol 86: 877–889. 10.1189/jlb.0708441 19498044

[38] Lin CC, Yu YL, Shih CC, Liu KJ, Ou KL, et al. (2011) A novel adjuvant Ling Zhi-8 enhances the efficacy of DNA cancer vaccine by activating dendritic cells. Cancer Immunol Immunother 60: 1019–1027. 10.1007/s00262-011-1016-4 21499904 PMC11029078

[39] Martinez-Montemayor MM, Acevedo RR, Otero-Franqui E, Cubano LA, Dharmawardhane SF (2011) Ganoderma lucidum (Reishi) inhibits cancer cell growth and expression of key molecules in inflammatory breast cancer. Nutr Cancer 63: 1085–1094. 10.1080/01635581.2011.601845 21888505 PMC3201987

[40] Liao CH, Hsiao YM, Hsu CP, Lin MY, Wang JC, et al. (2006) Transcriptionally mediated inhibition of telomerase of fungal immunomodulatory protein from Ganoderma tsugae in A549 human lung adenocarcinoma cell line. Mol Carcinog 45: 220–229. 10.1002/mc.20161 16402390

[41] Wu CT, Lin TY, Hsu HY, Sheu F, Ho CM, et al. (2011) Ling Zhi-8 mediates p53-dependent growth arrest of lung cancer cells proliferation via the ribosomal protein S7-MDM2-p53 pathway. Carcinogenesis 32: 1890–1896. 10.1093/carcin/bgr221 21983128

[42] Wang PH, Yang SF, Chen GD, Han CP, Chen SC, et al. (2007) Human nonmetastatic clone 23 type 1 gene suppresses migration of cervical cancer cells and enhances the migration inhibition of fungal immunomodulatory protein from Ganoderma tsugae. Reprod Sci 14: 475–485. 10.1177/1933719107305035 17913967

[43] Liao CH, Hsiao YM, Sheu GT, Chang JT, Wang PH, et al. (2007) Nuclear translocation of telomerase reverse transcriptase and calcium signaling in repression of telomerase activity in human lung cancer cells by fungal immunomodulatory protein from Ganoderma tsugae. Biochem Pharmacol 74: 1541–1554. 10.1016/j.bcp.2007.07.025 17720143

[44] Hsu HY, Hua KF, Wu WC, Hsu J, Weng ST, et al. (2008) Reishi immuno-modulation protein induces interleukin-2 expression via protein kinase-dependent signaling pathways within human T cells. J Cell Physiol 215: 15–26. 10.1002/jcp.21144 18189229

[45] Hu CT, Cheng CC, Pan SM, Wu JR, Wu WS (2013) PKC mediates fluctuant ERK-paxillin signaling for hepatocyte growth factor-induced migration of hepatoma cell HepG2. Cell Signal 25: 1457–1467. 10.1016/j.cellsig.2013.03.011 23524339

[46] Zhu AX, Gold PJ, El-Khoueiry AB, Abrams TA, Morikawa H, et al. (2013) First-in-man phase I study of GC33, a novel recombinant humanized antibody against glypican-3, in patients with advanced hepatocellular carcinoma. Clin Cancer Res 19: 920–928. 10.1158/1078-0432.CCR-12-2616 23362325

[47] Min L, He B, Hui L (2011) Mitogen-activated protein kinases in hepatocellular carcinoma development. Semin Cancer Biol 21: 10–20. 10.1016/j.semcancer.2010.10.011 20969960

[48] Kudo M (2012) Signaling pathway/molecular targets and new targeted agents under development in hepatocellular carcinoma. World J Gastroenterol 18: 6005–6017. 10.3748/wjg.v18.i42.6005 23155330 PMC3496878

[49] Zhang YH, Wang SQ, Sun CR, Wang M, Wang B, et al. (2011) Inhibition of JNK1 expression decreases migration and invasion of mouse hepatocellular carcinoma cell line in vitro. Med Oncol 28: 966–972. 10.1007/s12032-010-9568-2 20490718

[50] Radtke S, Milanovic M, Rosse C, De Rycker M, Lachmann S, et al. (2013) ERK2 but not ERK1 mediates HGF-induced motility in non-small cell lung carcinoma cell lines. J Cell Sci 126: 2381–2391. 10.1242/jcs.115832 23549785

[51] Chiang KC, Tsui KH, Chung LC, Yeh CN, Chen WT, et al. (2014) Celastrol blocks interleukin-6 gene expression via downregulation of NF-kappaB in prostate carcinoma cells. PLoS One 9: e93151 10.1371/journal.pone.0093151 24664372 PMC3963984

[52] Li T, Xu WS, Wu GS, Chen XP, Wang YT, et al. (2014) Platycodin D induces apoptosis, and inhibits adhesion, migration and invasion in HepG2 hepatocellular carcinoma cells. Asian Pac J Cancer Prev 15: 1745–1749. 24641402 10.7314/apjcp.2014.15.4.1745

[53] Fu WM, Zhang JF, Wang H, Xi ZC, Wang WM, et al. (2012) Heat shock protein 27 mediates the effect of 1,3,5-trihydroxy-13,13-dimethyl-2H-pyran [7,6-b] xanthone on mitochondrial apoptosis in hepatocellular carcinoma. J Proteomics 75: 4833–4843. 10.1016/j.jprot.2012.05.032 22677112

[54] Li N, Fu H, Tie Y, Hu Z, Kong W, et al. (2009) miR-34a inhibits migration and invasion by down-regulation of c-Met expression in human hepatocellular carcinoma cells. Cancer Lett 275: 44–53. 10.1016/j.canlet.2008.09.035 19006648

[55] Salvi A, Sabelli C, Moncini S, Venturin M, Arici B, et al. (2009) MicroRNA-23b mediates urokinase and c-met downmodulation and a decreased migration of human hepatocellular carcinoma cells. Febs J 276: 2966–2982. 10.1111/j.1742-4658.2009.07014.x 19490101

[56] Zhang SZ, Pan FY, Xu JF, Yuan J, Guo SY, et al. (2005) Knockdown of c-Met by adenovirus-delivered small interfering RNA inhibits hepatocellular carcinoma growth in vitro and in vivo. Mol Cancer Ther 4: 1577–1584. 10.1158/1535-7163.MCT-05-0106 16227408

[57] Abou-Alfa GK, Schwartz L, Ricci S, Amadori D, Santoro A, et al. (2006) Phase II study of sorafenib in patients with advanced hepatocellular carcinoma. J Clin Oncol 24: 4293–4300. 10.1200/JCO.2005.01.3441 16908937

[58] Villanueva A, Llovet JM (2011) Targeted therapies for hepatocellular carcinoma. Gastroenterology 140: 1410–1426. 10.1053/j.gastro.2011.03.006 21406195 PMC3682501

[59] Hagiwara S, Kudo M, Nagai T, Inoue T, Ueshima K, et al. (2012) Activation of JNK and high expression level of CD133 predict a poor response to sorafenib in hepatocellular carcinoma. Br J Cancer 106: 1997–2003. 10.1038/bjc.2012.145 22596232 PMC3388555

