# Supplementary figures and images for: Preclinical Trials for Prevention of Tumor Progression of Hepatocellular Carcinoma by LZ-8 Targeting c-Met Dependent and Independent Pathways
Source: PLoS One. 2015 Jan 21;10(1):e0114495. doi: 10.1371/journal.pone.0114495 (PMC4301873; doi:10.1371/journal.pone.0114495)

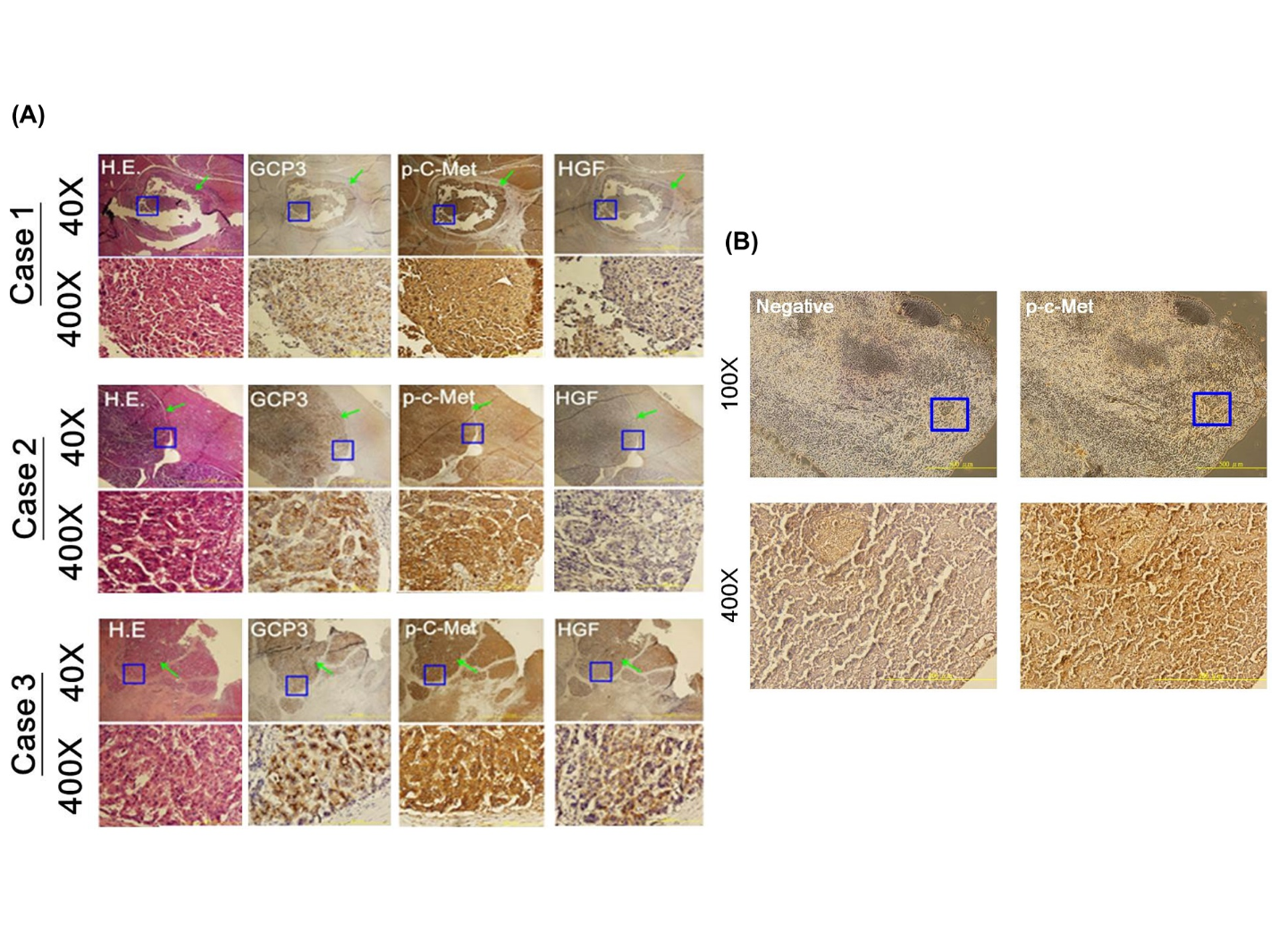

Supplement: S1 Fig — (A) IHC of the indicated molecules coupled with H & E stain was performed on tissue sections of three HCC cases. Green arrows indicate the location of tumor as verified by HCC tumor marker GPC3. The blue rectangle indicates the area that was magnified to 400×. The deep brown staining region revealed the location of indicated molecules in contrast to the light brown negative region. Data were representative of two reproducible experiments. (B) IHC of p-c-Met coupled with H & E stain was performed on tissues from which the indicated HCCs cell lines were derived. Imaging was performed through phase contrast microscopy. The image of negative controls (excluding the primary Ab incubation) was demonstrated in parallel. The area indicated by blue rectangles (100X magnification) was enlarged to 400X magnification. In the IHC of p-c-Met, the deep brown staining region revealed the location of p-c-Met in contrast to the light brown negative region. Data were representative of two reproducible experiments. (TIF) [file pone.0114495.s001.tif]

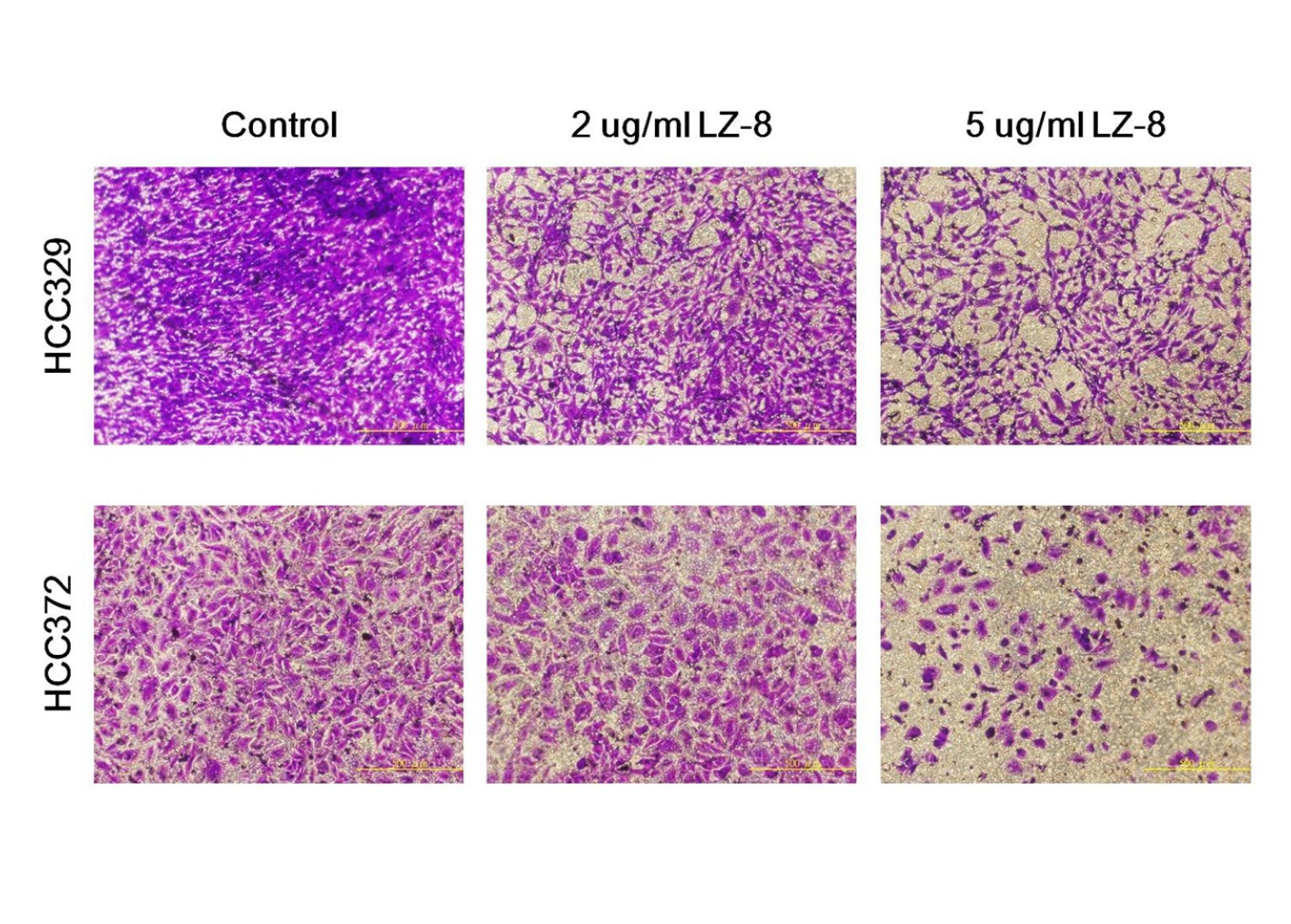

Supplement: S2 Fig — HCC372 and HCC329 were cultivated on a migration culture insert for 24 h and treated with LZ8 at indicated concentrations in a medium containing 10% serum for 24 h. Imaging was performed through phase contrast microscopy using 200× magnification. Data were representative of three reproducible experiments. (TIF) [file pone.0114495.s002.tif]

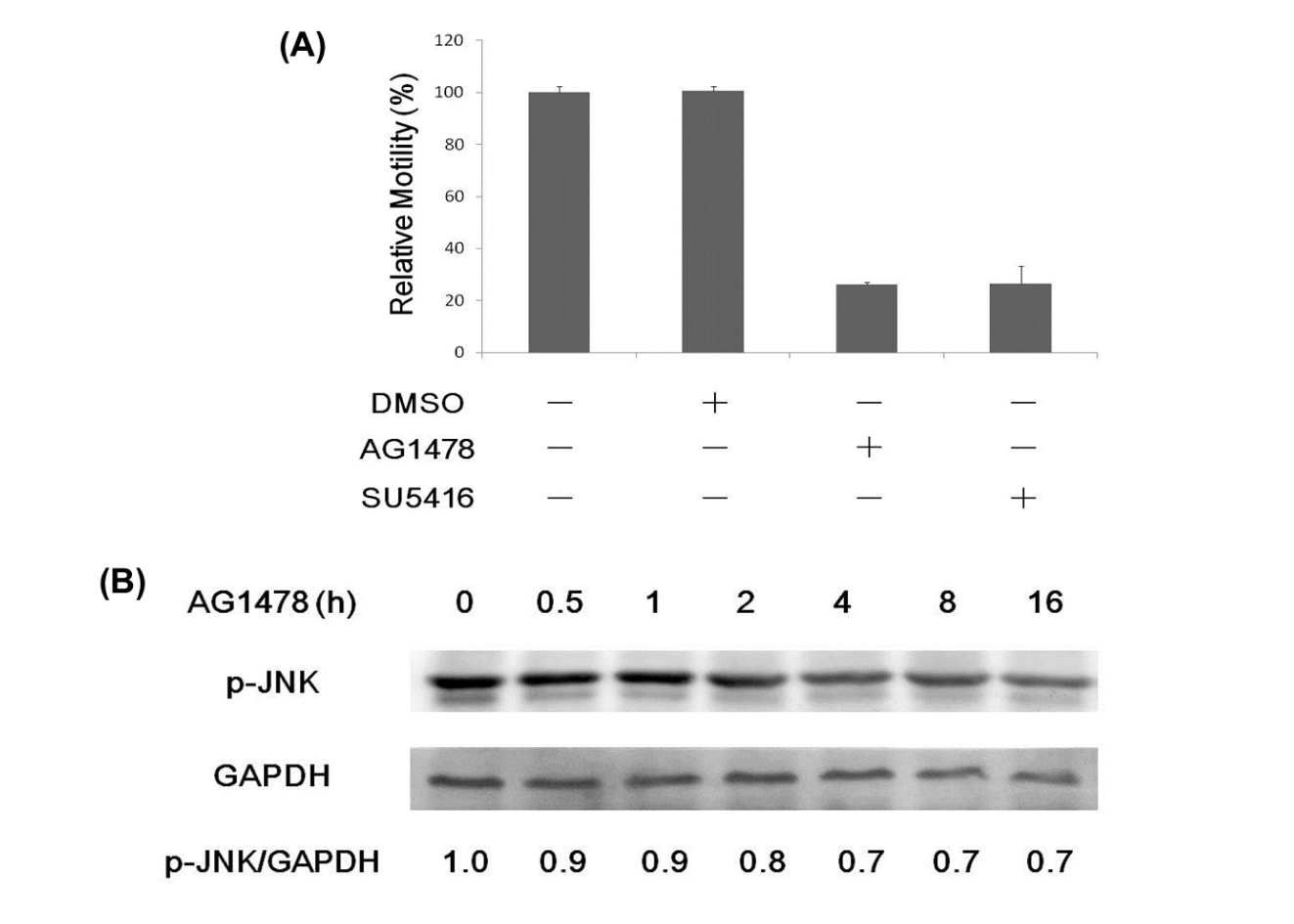

Supplement: S3 Fig — HCC329 cells were untreated (control) or treated with indicated inhibitors for 48 h (A) or indicated times (B). Wound healing migration analysis (A) and Western blot analysis of p-JNK (B) were performed. In (A), relative migration time was calculated, taking the data of control group as 100%. In (B), GAPDH was included as the loading control. The normalized intensity calculated as p-JNK/GAPDH is shown. Data were representative of three reproducible experiments. (TIF) [file pone.0114495.s003.tif]

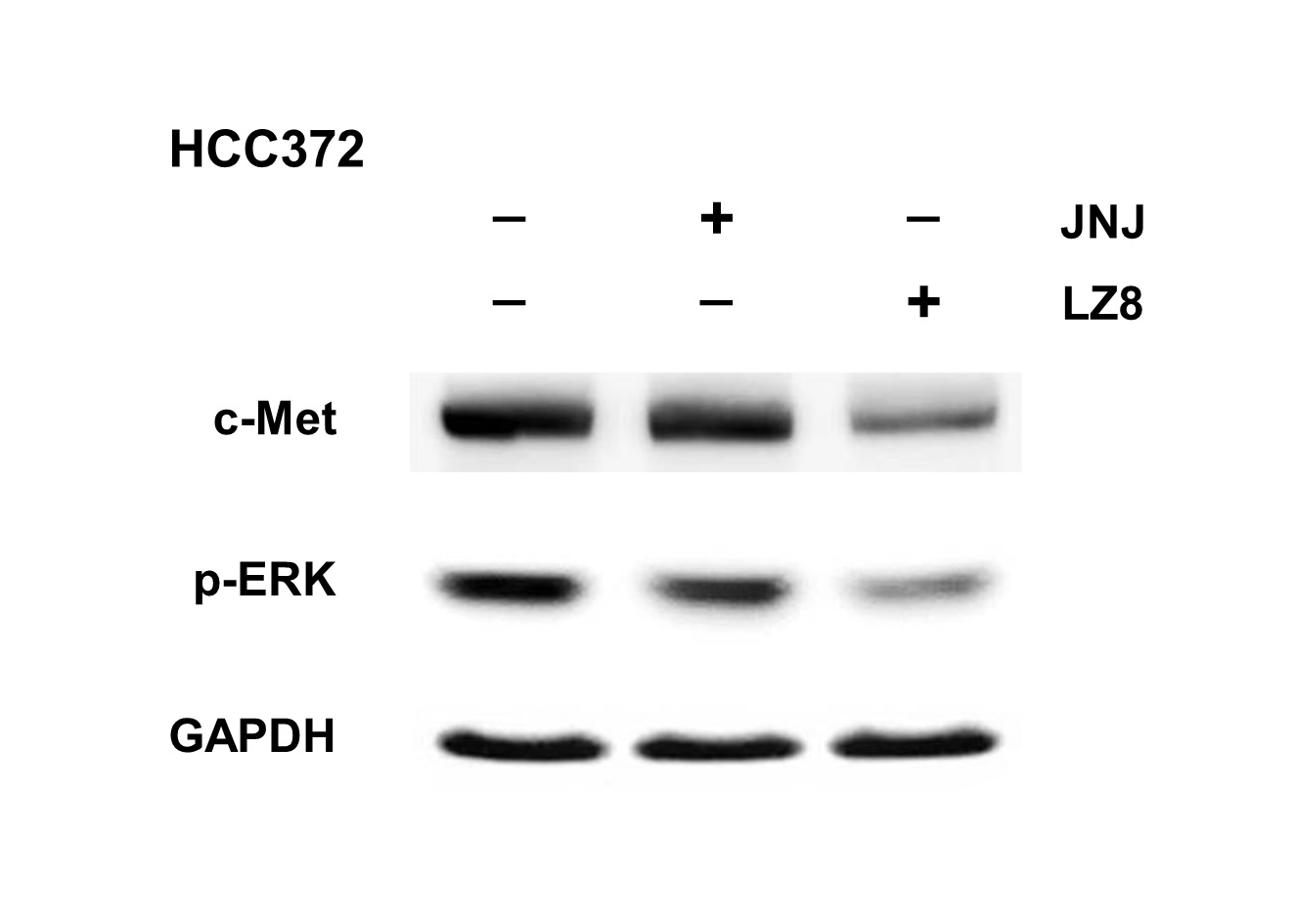

Supplement: S4 Fig — HCC372 cells were treated with JNJ (26.5 nM) or 2 μg/mL LZ8 for 4 h. Western blot of indicated signal molecules was performed using GAPDH as a loading control. Data were representative of three reproducible experiments. (TIF) [file pone.0114495.s004.tif]

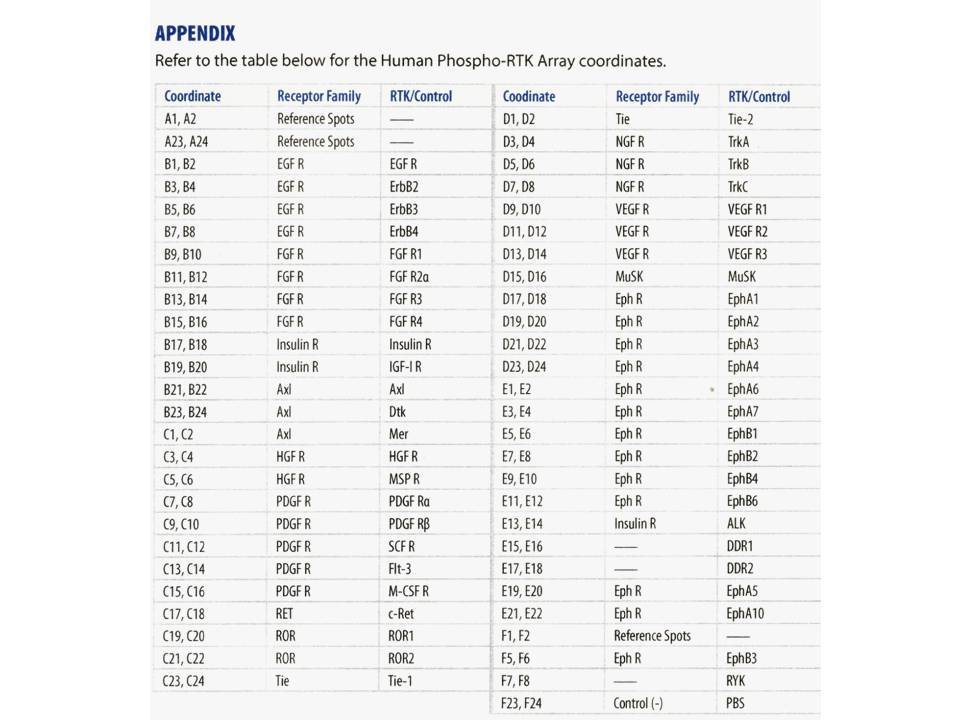

Supplement: S2 Materials — This is one of the appendixes in the manufacture’s protocol of Proteome Profile™ Array; R&D system. It provides the detailed information regarding the positions of the antibodies of 49 phosphorylated RTKs (p-RTKs) conjugated on the membrane for detecting the respective p-RTKs. The letters indicated in the most left (1st) and 4th column are the coordinates referring to the position of each antibody (in duplicate) indicated in the parallel column. (JPG) [file pone.0114495.s006.jpg]
