# Supplementary material for: Preclinical Trials for Prevention of Tumor Progression of Hepatocellular Carcinoma by LZ-8 Targeting c-Met Dependent and Independent Pathways
Source: PLoS One. 2015 Jan 21;10(1):e0114495. doi: 10.1371/journal.pone.0114495 (PMC4301873; doi:10.1371/journal.pone.0114495)
Supplement: S1 Materials — Signed informed consent forms (with partially hidden informations) of 4 HCC patients who agreed to provide their surgical HCC tissues for cell lines establishment in Liver Disease center in TZU CHI hospital Hualein, Taiwan. (PDF) [file pone.0114495.s005.pdf]

佛教慈濟綜合醫院

研究用人體檢體採集與使用同意書

民國100年11月29日研究倫理委員會會議修訂版

試驗計畫名稱：Paxillin及Hic-5在肝癌轉移中所扮演之角色

執行單位：花蓮、台中慈濟醫院及慈濟大學　　委託單位或經費來源：國科會

試驗主持人：胡志棠醫師　　職稱：肝膽腸胃科主任　　電話：03-8561825#5734

提供者姓名：曾 日 102/05/08 健 IC　U1 665321 男 024/12/12　0355 3613-1 一般外科　　出生年月日：

病歷號碼：[redacted] 聯絡電話：

通訊地址：[redacted]路1段201号

這份文件是為徵求您的同意，基於醫學研究發展需要，我們將採集您的檢體或收集您例行性醫療檢驗完畢後之剩餘檢體，做為往後進行相關研究使用。因此請您務必詳細閱讀以下的說明，以利於您做決定。您可以自由決定是否願意提供您的檢體進行研究，並可隨時撤銷此同意，不需任何理由，以上情況皆不會引起任何不愉快或影響日後我們對您的醫療照顧。

若您有任何疑問，可以詢問相關研究人員:胡志棠醫師及肝病研究中心(聯絡電話:03-8561825#5734)。

一、檢體取得之目的及可能之使用範圍、期間與地點

我們將進行肝癌復發與預後之肝內轉移分子研究，以臨床檢體建立之肝癌細胞株探討磷酸化的Paxillin與Hic-5在肝癌轉移中所扮演的角色，了解這兩個分子在肝癌轉移能力及預後的相關性、在細胞及動物實驗的肝癌細胞移動及侵犯情形、如何媒介訊息傳遞影響肝癌細胞移動及侵犯、並觀察在動物實驗肝內轉移是否被阻止。未來，Paxillin與Hic-5是否能作為防治肝內轉移之標靶將可被確認。因此請您提供檢體以助於未來的醫學發展。我們將保存您所提供之檢體並儲存於研究部肝病研究中心，直至西元二零一五年，期滿若仍未使用完畢、或您不同意將研究後剩餘之檢體提供未來使用，將依醫療廢棄物處理原則銷毀；在此期間，如果您欲撤除該剩餘檢體的使用權，且您的剩餘檢體尚未使用完畢，我們將銷毀您的剩餘檢體。

二、檢體採集之方法、部位、種類與數量

當您因疾病診斷或治療需要進行肝腫瘤切除手術或肝切片檢查時，為有助於將來肝癌疾病的防治，經過您的同意之後，我們將收集您於肝臟手術或肝切片時所切除取下的肝腫瘤及周邊正常組織，於常規進行切片送往病理科檢驗後的剩餘檢體，並將視剩餘情形將檢體分類、以直徑1公分以內分割為1-10片分裝，以供研究使用。

三、可能併發症或危險及預期之風險或不便

檢體採集時，我們將採集醫療行為完成後之剩餘組織檢體，您不會有任何併發症、危險性或副作用由於我們只使用您同意提供之檢體進行研究，因此不會給您帶來額外的風險或傷害。

四、檢體提供者之權益與檢體保管者、使用者之義務

您可以自由決定是否願意提供試驗結束後剩餘檢體以供未來的醫學研究，您也有權隨時撤除該剩餘檢體的使用權，任何決定都不會造成任何不愉快或影響日後我們對您的醫療照顧。如果您事後希望撤除該剩餘檢體的使用權，您可以隨時與我們聯絡（聯絡人：胡志棠醫師、單位：肝膽腸胃科、研究部肝病研究中心 電話：03-8561825 分機 5734）。

慈濟醫院(以下簡稱本院)或經慈濟醫院同意的研究者若要使用您的檢體進行其他醫學研究，必須先提出研究計畫書，並經本院研究倫理委員會就研究之目的、重要性、檢體使用方式、保密措施等項目，審核檢體使用的適當性，經審核同意後才能使用您的檢體，以求保障您的權益。

若您對自身權益有疑問可與本院之研究倫理委員會聯絡請求諮詢，電話：03-8561825 分機 2124、傳真：03-8561825 分機 3272。

五、預期之研究成果

由於未來的研究成果目前尚難以得知，您個人極有可能不會因研究結果而獲得任何醫療上的利益。研究結果若因此產生學術文獻發表、實質效益或衍生其他權益時，您亦同意將依法回饋使用於疾病預防、診斷及治療等公益用途。

六、保障檢體提供者個人隱私的機制

我們將以一組編號來取代您的姓名、身份證字號或病歷號碼等任何可以連結到您個人資料的代碼來標示您的檢體，只有本院研究者及其授權的研究人員有權使用您的檢體及與您相關之健康資訊。胡志棠醫師及其研究團隊將依法把任何可辨識您身分的個人資料視為機密來處理，不會公開。研究所得資料可能發表於學術性雜誌，但不會公佈您的姓名或可辨識您身分的個人資料，您的個人隱私將給予絕對之保密。

七、研究檢體所得資訊對檢體提供者及其親屬或族群可能造成的影響

我們無法預知未來研究結果是否對您及您的家屬或族群的健康造成任何影響。研究結果若有任何與您們的健康有關的重大資訊，我們將尊重您的選擇而決定是否將此資訊提供給您本人，再由您決定是否同意我們將相關資訊提供給您的家屬。其他與您族群健康有關的重大資訊，我們將會慎重考量公佈此研究結果的方式，並依相關規定辦理。

八、檢體是否提供、讓與或授權本院以外之他人使用

有關您的檢體是否有提供、讓與或授權機構外之他人使用，將由慈濟醫院研究倫理委員會審查其適當性，以保障您的權益。

九、研究經費來源及所有參與研究之機構

您的檢體若用於未來的醫學研究，目前無法知道該研究的經費來源及其他參與研究之機構，此部分將由慈濟醫院研究倫理委員會於未來審查過程中代為瞭解。

十、試驗成果及權益歸屬

如本試驗研究成果產生學術文獻發表、智慧財產及其他效益時，受試者同意無償捐贈給本院從事疾病預防、診斷及治療等公益用途。

十一、其他依各研究計畫之需要，與檢體採集、病歷檢閱、追蹤檢查檢驗或病情資訊相關之重要事項

本院研究者、研究贊助者、本院研究倫理委員會與衛生主管單位皆有權依各研究計畫之需要檢視您的病歷、檢查結果或病情資訊等相關事項，並遵守保密之義務。

說明醫師簽名：[illegible] 日期：102 年 5 月 16 日

1. 您可以自由決定是否同意提供檢體供未來醫學研究

☐ 我同意提供檢體供此次研究使用，若未來有任何的研究需使用我的檢體，我同意授權慈濟醫院研究倫理委員會審核使用本人檢體之適當性。

☐ 我同意提供檢體供此次研究使用，若未來有任何的研究需使用我的檢體，每次皆應徵求我的同意；除非檢體已經編碼且與我個人資料永久去連結。

☐ 我僅同意提供檢體供此次研究使用，研究結束後請將檢體銷毀。

2. 您是否希望知道任何由此檢體研究而得知與您或您的家人健康有重大關聯的研究結果

我本人 ☑ 希望 ☐ 不希望 被通知

我家人 ☐ 希望 ☐ 不希望 被通知

立同意書人簽名：曾[redacted]日 日期：102 年 5 月 16 日

法定代理人或有同意權人簽名：__________ 日期：____ 年 ____ 月 ____ 日

與病患之關係(請圈選)：配偶、父、母、兒、女、其他：__________

住址：__________ 電話：__________

＊ 受試者為無行為能力(未滿七歲之未成年人者或受監護宣告之人)，由法定代理人為之；受試者為限制行為人者(滿七歲以上之未成年人或受輔助宣告之人)，應得本人及其法定代理人之同意。

＊ 受試者雖非無行為能力或限制行為能力者，但因意識混亂或有精神與智能障礙，而無法進行有效 溝通和判斷時由有同意權之人為之。前項有同意權人為配偶及同居之親屬。

若病人意識不清且無親屬或關係人在場，需有見證人在場，見證同意書之取得過程。試驗相關人員不得為見證人。

見證人姓名： 身份證字號： 聯絡電話：

通訊地址：

簽名：__________ 日期：____ 年 ____ 月 ____ 日

佛教慈濟綜合醫院
研究用人體檢體採集與使用同意書

民國 100 年 11 月 29 日研究倫理委員會會議修訂版

試驗計畫名稱：Paxillin及Hic-5在肝癌轉移中所扮演之角色

執行單位：花蓮、台中慈濟醫院及慈濟大學　　**委託單位或經費來源：**國科會

試驗主持人：胡志棠醫師　　**職稱：**肝膽腸胃科主任　　**電話：**03-8561825#5734

提供者姓名： 宋[redacted]閩　103/03/18建檔　　**出生年月日：**
U1[redacted]538281 男 047/01/20
0355 3602-1　一般外科
病歷號碼：　　**聯絡電話：**

通訊地址：[redacted]

這份文件是為徵求您的同意，基於醫學研究發展需要，我們將採集您的檢體或收集您例行性醫療檢驗完畢後之剩餘檢體，做為往後進行相關研究使用。因此請您務必詳細閱讀以下的說明，以利於您做決定。您可以自由決定是否願意提供您的檢體進行研究，並可隨時撤銷此同意，不需任何理由，以上情況皆不會引起任何不愉快或影響日後我們對您的醫療照顧。

若您有任何疑問，可以詢問相關研究人員:胡志棠醫師及肝病研究中心(聯絡電話:03-8561825#5734)。

一、檢體取得之目的及可能之使用範圍、期間與地點

我們將進行肝癌復發與預後之肝內轉移分子研究，以臨床檢體建立之肝癌細胞株探討磷酸化的 Paxillin 與Hic-5在肝癌轉移中所扮演的角色，了解這兩個分子在肝癌轉移能力及預後的相關性、在細胞及動物實驗的肝癌細胞移動及侵犯情形、如何媒介訊息傳遞影響肝癌細胞移動及侵犯、並觀察在動物實驗肝內轉移是否被阻止。未來，Paxillin 與Hic-5是否能作為防治肝內轉移之標靶將可被確認。因此請您提供檢體以助於未來的醫學發展。我們將保存您所提供之檢體並儲存於研究部肝病研究中心，直至西元二零一五年，期滿若仍未使用完畢、或您不同意將研究後剩餘之檢體提供未來使用，將依醫療廢棄物處理原則銷毀；在此期間，如果您欲撤除該剩餘檢體的使用權，且您的剩餘檢體尚未使用完畢，我們將銷毀您的剩餘檢體。

二、檢體採集之方法、部位、種類與數量

當您因疾病診斷或治療需要進行肝腫瘤切除手術或肝切片檢查時，為有助於將來肝癌疾病的防治，經過您的同意之後，我們將收集您於肝臟手術或肝切片時所切除取下的肝腫瘤及周邊正常組織，於常規進行切片送往病理科檢驗後的剩餘檢體，並將視剩餘情形將檢體分類、以直徑 1 公分以內分割為 1-10 片分裝，以供研究使用。

三、可能併發症或危險及預期之風險或不便

檢體採集時，我們將採集醫療行為完成後之剩餘組織檢體，您不會有任何併發症、危險性或副作用由於我們只使用您同意提供之檢體進行研究，因此不會給您帶來額外的風險或傷害。

四、檢體提供者之權益與檢體保管者、使用者之義務

您可以自由決定是否願意提供試驗結束後剩餘檢體以供未來的醫學研究，您也有權隨時撤除該剩餘檢體的使用權，任何決定都不會造成任何不愉快或影響日後我們對您的醫療照顧。如果您事後希望撤除該剩餘檢體的使用權，您可以隨時與我們聯絡（聯絡人：胡志棠醫師、單位：肝膽腸胃科、研究部肝病研究中心 電話：03-8561825 分機 5734）。

慈濟醫院(以下簡稱本院)或經慈濟醫院同意的研究者若要使用您的檢體進行其他醫學研究，必須先提出研究計畫書，並經本院研究倫理委員會就研究之目的、重要性、檢體使用方式、保密措施等項目，審核檢體使用的適當性，經審核同意後才能使用您的檢體，以求保障您的權益。

若您對自身權益有疑問可與本院之研究倫理委員會聯絡請求諮詢，電話：03-8561825 分機 2124、傳真：03-8561825 分機 3272。

五、預期之研究成果

由於未來的研究成果目前尚難以得知，您個人極有可能不會因研究結果而獲得任何醫療上的利益。研究結果若因此產生學術文獻發表、實質效益或衍生其他權益時，您亦同意將依法回饋使用於疾病預防、診斷及治療等公益用途。

六、保障檢體提供者個人隱私的機制

我們將以一組編號來取代您的姓名、身份證字號或病歷號碼等任何可以連結到您個人資料的代碼來標示您的檢體，只有本院研究者及其授權的研究人員有權使用您的檢體及與您相關之健康資訊。
胡志棠醫師及其研究團隊將依法把任何可辨識您身分的個人資料視為機密來處理，不會公開。研究所得資料可能發表於學術性雜誌，但不會公佈您的姓名或可辨識您身分的個人資料，您的個人隱私將給予絕對之保密。

七、研究檢體所得資訊對檢體提供者及其親屬或族群可能造成的影響

我們無法預知未來研究結果是否對您及您的家屬或族群的健康造成任何影響。研究結果若有任何與您們的健康有關的重大資訊，我們將尊重您的選擇而決定是否將此資訊提供給您本人，再由您決定是否同意我們將相關資訊提供給您的家屬。其他與您族群健康有關的重大資訊，我們將會慎重考量公佈此研究結果的方式，並依相關規定辦理。

八、檢體是否提供、讓與或授權本院以外之他人使用

有關您的檢體是否有提供、讓與或授權機構外之他人使用，將由慈濟醫院研究倫理委員會審查其適當性，以保障您的權益。

九、研究經費來源及所有參與研究之機構

您的檢體若用於未來的醫學研究，目前無法知道該研究的經費來源及其他參與研究之機構，此部分將由慈濟醫院研究倫理委員會於未來審查過程中代為瞭解。

十、試驗成果及權益歸屬

如本試驗研究成果產生學術文獻發表、智慧財產及其他效益時，受試者同意無償捐贈給本院從事疾病預防、診斷及治療等公益用途。

十一、其他依各研究計畫之需要，與檢體採集、病歷檢閱、追蹤檢查檢驗或病情資訊相關之重要事項

本院研究者、研究贊助者、本院研究倫理委員會與衛生主管單位皆有權依各研究計畫之需要檢視您的病歷、檢查結果或病情資訊等相關事項，並遵守保密之義務。

說明醫師簽名：李明杰　日期：103 年 5 月 5 日

1. 您可以自由決定是否同意提供檢體供未來醫學研究

☑ 我同意提供檢體供此次研究使用，若未來有任何的研究需使用我的檢體，我同意授權慈濟醫院研究倫理委員會審核使用本人檢體之適當性。

☐ 我同意提供檢體供此次研究使用，若未來有任何的研究需使用我的檢體，每次皆應徵求我的同意；除非檢體已經編碼且與我個人資料永久去連結。

☐ 我僅同意提供檢體供此次研究使用，研究結束後請將檢體銷毀。

2. 您是否希望知道任何由此檢體研究而得知與您或您的家人健康有重大關聯的研究結果

我本人 ☑ 希望 ☐ 不希望 被通知

我家人 ☑ 希望 ☐ 不希望 被通知

立同意書人簽名：宋[redacted]潤　日期：103 年 5 月 5 日

法定代理人或有同意權人簽名：＿＿＿＿＿＿日期：＿＿＿＿ 年＿＿＿月＿＿＿日

與病患之關係（請圈選）：配偶、父、母、兒、女、其他：＿＿＿＿＿＿

住址：花市中[redacted]　電話：03-8[redacted]10

＊ 受試者為無行為能力(未滿七歲之未成年人者或受監護宣告之人)，由法定代理人為之；受試者為限制行為人者(滿七歲以上之未成年人或受輔助宣告之人)，應得本人及其法定代理人之同意。

＊ 受試者雖非無行為能力或限制行為能力者，但因意識混亂或有精神與智能障礙，而無法進行有效 溝通和判斷時由有同意權之人為之。前項有同意權人為配偶及同居之親屬。

若病人意識不清且無親屬或關係人在場，需有見證人在場，見證同意書之取得過程。試驗相關人員不得為見證人。

見證人姓名：　身份證字號：　聯絡電話：

通訊地址：

簽名：＿＿＿＿＿＿　日期：＿＿＿＿ 年＿＿＿月＿＿＿日

佛教慈濟綜合醫院

研究用人體檢體採集與使用同意書

民國100年11月29日研究倫理委員會會議修訂版

試驗計畫名稱：Paxillin及Hic-5在肝癌轉移中所扮演之角色

執行單位：花蓮、台中慈濟醫院及慈濟大學　　委託單位或經費來源：國科會

試驗主持人：胡志棠醫師　　職稱：肝膽腸胃科主任　　電話：03-8561825#5734

提供者姓名：黃　賢　103/04/29　　出生年月日：

U2 456440 女 045/02/22

病歷號碼：0355 3611-3 一般外科

通訊地址：[redacted]

這份文件是為徵求您的同意，基於醫學研究發展需要，我們將採集您的檢體或收集您例行性醫療檢驗完畢後之剩餘檢體，做為往後進行相關研究使用。因此請您務必詳細閱讀以下的說明，以利於您做決定。您可以自由決定是否願意提供您的檢體進行研究，並可隨時撤銷此同意，不需任何理由，以上情況皆不會引起任何不愉快或影響日後我們對您的醫療照顧。

若您有任何疑問，可以詢問相關研究人員:胡志棠醫師及肝病研究中心(聯絡電話: 03-8561825#5734)。

一、檢體取得之目的及可能之使用範圍、期間與地點

我們將進行肝癌復發與預後之肝內轉移分子研究，以臨床檢體建立之肝癌細胞株探討磷酸化的Paxillin 與Hic-5在肝癌轉移中所扮演的角色，了解這兩個分子在肝癌轉移能力及預後的相關性、在細胞及動物實驗的肝癌細胞移動及侵犯情形、如何媒介訊息傳遞影響肝癌細胞移動及侵犯、並觀察在動物實驗肝內轉移是否被阻止。未來，Paxillin 與Hic-5是否能作為防治肝內轉移之標靶將可被確認。因此請您提供檢體以助於未來的醫學發展。我們將保存您所提供之檢體並儲存於研究部肝病研究中心，直至西元二零一五年，期滿若仍未使用完畢、或您不同意將研究後剩餘之檢體提供未來使用，將依醫療廢棄物處理原則銷毀；在此期間，如果您欲撤除該剩餘檢體的使用權，且您的剩餘檢體尚未使用完畢，我們將銷毀您的剩餘檢體。

二、檢體採集之方法、部位、種類與數量

當您因疾病診斷或治療需要進行肝腫瘤切除手術或肝切片檢查時，為有助於將來肝癌疾病的防治，經過您的同意之後，我們將收集您於肝臟手術或肝切片時所切除取下的肝腫瘤及周邊正常組織，於常規進行切片送往病理科檢驗後的剩餘檢體，並將視剩餘情形將檢體分類、以直徑1公分以內分割為1-10片分裝，以供研究使用。

三、可能併發症或危險及預期之風險或不便

檢體採集時，我們將採集醫療行為完成後之剩餘組織檢體，您不會有任何併發症、危險性或副作用由於我們只使用您同意提供之檢體進行研究，因此不會給您帶來額外的風險或傷害。

四、檢體提供者之權益與檢體保管者、使用者之義務

您可以自由決定是否願意提供試驗結束後剩餘檢體以供未來的醫學研究，您也有權隨時撤除該剩餘檢體的使用權，任何決定都不會造成任何不愉快或影響日後我們對您的醫療照顧。如果您事後希望撤除該剩餘檢體的使用權，您可以隨時與我們聯絡（聯絡人：胡志棠醫師、單位：肝膽腸胃科、研究部肝病研究中心 電話：03-8561825 分機 5734）。

慈濟醫院(以下簡稱本院)或經慈濟醫院同意的研究者若要使用您的檢體進行其他醫學研究，必須先提出研究計畫書，並經本院研究倫理委員會就研究之目的、重要性、檢體使用方式、保密措施等項目，審核檢體使用的適當性，經審核同意後才能使用您的檢體，以求保障您的權益。

若您對自身權益有疑問可與本院之研究倫理委員會聯絡請求諮詢，電話：03-8561825 分機 2124、傳真：03-8561825 分機 3272。

五、預期之研究成果

由於未來的研究成果目前尚難以得知，您個人極有可能不會因研究結果而獲得任何醫療上的利益。研究結果若因此產生學術文獻發表、實質效益或衍生其他權益時，您亦同意將依法回饋使用於疾病預防、診斷及治療等公益用途。

六、保障檢體提供者個人隱私的機制

我們將以一組編號來取代您的姓名、身份證字號或病歷號碼等任何可以連結到您個人資料的代碼來標示您的檢體，只有本院研究者及其授權的研究人員有權使用您的檢體及與您相關之健康資訊。胡志棠醫師及其研究團隊將依法把任何可辨識您身分的個人資料視為機密來處理，不會公開。研究所得資料可能發表於學術性雜誌，但不會公佈您的姓名或可辨識您身分的個人資料，您的個人隱私將給予絕對之保密。

七、研究檢體所得資訊對檢體提供者及其親屬或族群可能造成的影響

我們無法預知未來研究結果是否對您及您的家屬或族群的健康造成任何影響。研究結果若有任何與您們的健康有關的重大資訊，我們將尊重您的選擇而決定是否將此資訊提供給您本人，再由您決定是否同意我們將相關資訊提供給您的家屬。其他與您族群健康有關的重大資訊，我們將會慎重考量公佈此研究結果的方式，並依相關規定辦理。

八、檢體是否提供、讓與或授權本院以外之他人使用

有關您的檢體是否有提供、讓與或授權機構外之他人使用，將由慈濟醫院研究倫理委員會審查其適當性，以保障您的權益。

九、研究經費來源及所有參與研究之機構

您的檢體若用於未來的醫學研究，目前無法知道該研究的經費來源及其他參與研究之機構，此部分將由慈濟醫院研究倫理委員會於未來審查過程中代為瞭解。

十、試驗成果及權益歸屬

如本試驗研究成果產生學術文獻發表、智慧財產及其他效益時，受試者同意無償捐贈給本院從事疾病預防、診斷及治療等公益用途。

十一、其他依各研究計畫之需要，與檢體採集、病歷檢閱、追蹤檢查檢驗或病情資訊相關之重要事項
本院研究者、研究贊助者、本院研究倫理委員會與衛生主管單位皆有權依各研究計畫之需要檢視您的病歷、檢查結果或病情資訊等相關事項，並遵守保密之義務。

說明醫師簽名：＿＿[signature]＿＿ 日期：＿103＿年＿4＿月＿29＿日

1. 您可以自由決定是否同意提供檢體供未來醫學研究

☑ **我同意提供檢體供此次研究使用，若未來有任何的研究需使用我的檢體，我同意授權慈濟醫院研究倫理委員會審核使用本人檢體之適當性。**

☐ **我同意提供檢體供此次研究使用，若未來有任何的研究需使用我的檢體，每次皆應徵求我的同意；除非檢體已經編碼且與我個人資料永久去連結。**

☐ **我僅同意提供檢體供此次研究使用，研究結束後請將檢體銷毀。**

2. 您是否希望知道任何由此檢體研究而得知與您或您的家人健康有重大關聯的研究結果

我本人 ☐ 希望 ☑ 不希望 被通知

我家人 ☐ 希望 ☑ 不希望被通知

立同意書人簽名：＿黃[redacted]香＿ 日期：＿103＿年＿4＿月＿29＿日

法定代理人或有同意權人簽名：＿徐[redacted]濟＿ 日期：＿103＿年＿4＿月＿29＿日

與病患之關係（請圈選）：配偶、父、母、子、女、其他：＿＿＿＿

住址：花縣新城鄉[redacted] 電話：[redacted]6

＊ 受試者為無行為能力(未滿七歲之未成年人者或受監護宣告之人)，由法定代理人為之；受試者為限制行為人者(滿七歲以上之未成年人或受輔助宣告之人)，應得本人及其法定代理人之同意。

＊ 受試者雖非無行為能力或限制行為能力者，但因意識混亂或有精神與智能障礙，而無法進行有效 溝通和判斷時由有同意權之人為之。前項有同意權人為配偶及同居之親屬。

若病人意識不清且無親屬或關係人在場，需有見證人在場，見證同意書之取得過程。試驗相關人員不得為見證人。

見證人姓名： 身份證字號： 聯絡電話：

通訊地址：

簽名：＿＿＿＿＿＿ 日期：＿＿＿＿年＿＿＿月＿＿＿日

PH010

佛教慈濟綜合醫院

研究用人體檢體採集與使用同意書

傅[redacted]和 103/06/09鍾佑
V1[redacted]036661 男 032/10/04
0355 3707-1 一般外科

民國100年11月29日研究倫理委員會會議修訂版

試驗計畫名稱：Paxillin及Hic-5在肝癌轉移中所扮演之角色

執行單位：花蓮、台中慈濟醫院及慈濟大學　委託單位或經費來源：國科會

試驗主持人：胡志棠醫師　職稱：肝膽腸胃科主任　電話：03-8561825#5734

提供者姓名：　性別：　出生年月日：

病歷號碼：[redacted]　聯絡電話：

通訊地址：

這份文件是為徵求您的同意，基於醫學研究發展需要，我們將採集您的檢體或收集您例行性醫療檢驗完畢後之剩餘檢體，做為往後進行相關研究使用。因此請您務必詳細閱讀以下的說明，以利於您做決定。您可以自由決定是否願意提供您的檢體進行研究，並可隨時撤銷此同意，不需任何理由，以上情況皆不會引起任何不愉快或影響日後我們對您的醫療照顧。

若您有任何疑問，可以詢問相關研究人員:胡志棠醫師及肝病研究中心(聯絡電話:03-8561825#5734)。

一、檢體取得之目的及可能之使用範圍、期間與地點

我們將進行肝癌復發與預後之肝內轉移分子研究，以臨床檢體建立之肝癌細胞株探討磷酸化的Paxillin與Hic-5在肝癌轉移中所扮演的角色，了解這兩個分子在肝癌轉移能力及預後的相關性、在細胞及動物實驗的肝癌細胞移動及侵犯情形、如何媒介訊息傳遞影響肝癌細胞移動及侵犯、並觀察在動物實驗肝內轉移是否被阻止。未來，Paxillin與Hic-5是否能作為防治肝內轉移之標靶將可被確認。因此請您提供檢體以助於未來的醫學發展。我們將保存您所提供之檢體並儲存於研究部肝病研究中心，直至西元二零一五年，期滿若仍未使用完畢、或您不同意將研究後剩餘之檢體提供未來使用，將依醫療廢棄物處理原則銷毀；在此期間，如果您欲撤除該剩餘檢體的使用權，且您的剩餘檢體尚未使用完畢，我們將銷毀您的剩餘檢體。

二、檢體採集之方法、部位、種類與數量

當您因疾病診斷或治療需要進行肝腫瘤切除手術或肝切片檢查時，為有助於將來肝癌疾病的防治，經過您的同意之後，我們將收集您於肝臟手術或肝切片時所切除取下的肝腫瘤及周邊正常組織，於常規進行切片送往病理科檢驗後的剩餘檢體，並將視剩餘情形將檢體分類、以直徑1公分以內分割為1-10片分裝，以供研究使用。

三、可能併發症或危險及預期之風險或不便

檢體採集時，我們將採集醫療行為完成後之剩餘組織檢體，您不會有任何併發症、危險性或副作用由於我們只使用您同意提供之檢體進行研究，因此不會給您帶來額外的風險或傷害。

四、檢體提供者之權益與檢體保管者、使用者之義務

您可以自由決定是否願意提供試驗結束後剩餘檢體以供未來的醫學研究，您也有權隨時撤除該剩餘檢體的使用權，任何決定都不會造成任何不愉快或影響日後我們對您的醫療照顧。如果您事後希望撤除該剩餘檢體的使用權，您可以隨時與我們聯絡（聯絡人：胡志棠醫師、單位：肝膽腸胃科、研究部肝病研究中心 電話：03-8561825 分機 5734）。

慈濟醫院(以下簡稱本院)或經慈濟醫院同意的研究者若要使用您的檢體進行其他醫學研究，必須先提出研究計畫書，並經本院研究倫理委員會就研究之目的、重要性、檢體使用方式、保密措施等項目，審核檢體使用的適當性，經審核同意後才能使用您的檢體，以求保障您的權益。

若您對自身權益有疑問可與本院之研究倫理委員會聯絡請求諮詢，電話：03-8561825 分機 2124、傳真：03-8561825 分機 3272。

五、預期之研究成果

由於未來的研究成果目前尚難以得知，您個人極有可能不會因研究結果而獲得任何醫療上的利益。研究結果若因此產生學術文獻發表、實質效益或衍生其他權益時，您亦同意將依法回饋使用於疾病預防、診斷及治療等公益用途。

六、保障檢體提供者個人隱私的機制

我們將以一組編號來取代您的姓名、身份證字號或病歷號碼等任何可以連結到您個人資料的代碼來標示您的檢體，只有本院研究者及其授權的研究人員有權使用您的檢體及與您相關之健康資訊。胡志棠醫師及其研究團隊將依法把任何可辨識您身分的個人資料視為機密來處理，不會公開。研究所得資料可能發表於學術性雜誌，但不會公佈您的姓名或可辨識您身分的個人資料，您的個人隱私將給予絕對之保密。

七、研究檢體所得資訊對檢體提供者及其親屬或族群可能造成的影響

我們無法預知未來研究結果是否對您及您的家屬或族群的健康造成任何影響。研究結果若有任何與您們的健康有關的重大資訊，我們將尊重您的選擇而決定是否將此資訊提供給您本人，再由您決定是否同意我們將相關資訊提供給您的家屬。其他與您族群健康有關的重大資訊，我們將會慎重考量公佈此研究結果的方式，並依相關規定辦理。

八、檢體是否提供、讓與或授權本院以外之他人使用

有關您的檢體是否有提供、讓與或授權機構外之他人使用，將由慈濟醫院研究倫理委員會審查其適當性，以保障您的權益。

九、研究經費來源及所有參與研究之機構

您的檢體若用於未來的醫學研究，目前無法知道該研究的經費來源及其他參與研究之機構，此部分將由慈濟醫院研究倫理委員會於未來審查過程中代為瞭解。

十、試驗成果及權益歸屬

如本試驗研究成果產生學術文獻發表、智慧財產及其他效益時，受試者同意無償捐贈給本院從事疾病預防、診斷及治療等公益用途。

十一、其他依各研究計畫之需要，與檢體採集、病歷檢閱、追蹤檢查檢驗或病情資訊相關之重要事項
本院研究者、研究贊助者、本院研究倫理委員會與衛生主管單位皆有權依各研究計畫之需要檢視您的病歷、檢查結果或病情資訊等相關事項，並遵守保密之義務。

說明醫師簽名：[illegible] 日期：103 年 6 月 9 日

1. 您可以自由決定是否同意提供檢體供未來醫學研究

☐ 我同意提供檢體供此次研究使用，若未來有任何的研究需使用我的檢體，我同意授權慈濟醫院研究倫理委員會審核使用本人檢體之適當性。

☐ 我同意提供檢體供此次研究使用，若未來有任何的研究需使用我的檢體，每次皆應徵求我的同意；除非檢體已經編碼且與我個人資料永久去連結。

☐ 我僅同意提供檢體供此次研究使用，研究結束後請將檢體銷毀。

2. 您是否希望知道任何由此檢體研究而得知與您或您的家人健康有重大關聯的研究結果

我本人 ☐ 希望 ☑ 不希望 被通知

我家人 ☐ 希望 ☑ 不希[illegible]通知

立同意書人簽名：傅[illegible]偉 日期：103 年 6 月 9 日

法定代理人或有同意權人簽名：＿＿＿＿＿ 日期：＿＿＿ 年＿＿＿ 月＿＿＿ 日

與病患之關係（請圈選[illegible]、其他：＿＿＿＿

住址：台東市[illegible] 電話：089[illegible]21

* 受試者為無行為能力(未滿七歲之未成年人者或受監護宣告之人)，由法定代理人為之；受試者為限制行為人者(滿七歲以上之未成年人或受輔助宣告之人)，應得本人及其法定代理人之同意。

* 受試者雖非無行為能力或限制行為能力者，但因意識混亂或有精神與智能障礙，而無法進行有效 溝通和判斷時由有同意權之人為之。前項有同意權人為配偶及同居之親屬。

若病人意識不清且無親屬或關係人在場，需有見證人在場，見證同意書之取得過程。試驗相關人員不得為見證人。

見證人姓名： 身份證字號： 聯絡電話：

通訊地址：

簽名：＿＿＿＿＿ 日期：＿＿＿ 年＿＿＿ 月＿＿＿ 日
