# Supplementary material for: Preclinical Trials for Prevention of Tumor Progression of Hepatocellular Carcinoma by LZ-8 Targeting c-Met Dependent and Independent Pathways
Source: PLoS One. 2015 Jan 21;10(1):e0114495. doi: 10.1371/journal.pone.0114495 (PMC4301873; doi:10.1371/journal.pone.0114495)
Supplement: S3 Materials — The copied certificate of English editing for this paper by a mother tongue English speaker, Dr. Jennifer Sampson in Wallace Academic Editing Company. (PDF) [file pone.0114495.s007.pdf]

Wallace Academic Editing

English Editing Certificate

This certifies that the paper **Preclinical trials for prevention of tumor progression of hepatocellular carcinoma by LZ-8 targeting c-Met dependent and independent pathways** has been edited by Jennifer Sampson on October 25th, 2014 and is considered to be improved in grammar, punctuation, spelling, verb usage, sentence structure, conciseness, general readability, writing style, and native English usage to the best of the editor's ability.

Best regards,
Wallace Academic Editing

Phone No.: +886-2-2555-5830
Website: http://www.editing.tw
Email: editing@wallace.tw
Address: 3F., No.180, Chang'an W. Rd., Datong Dist., Taipei City
